# Recent Advances on the Fabrication of Antifouling Phase-Inversion Membranes by Physical Blending Modification Method

**DOI:** 10.3390/membranes13010058

**Published:** 2023-01-02

**Authors:** Tesfaye Abebe Geleta, Irish Valerie Maggay, Yung Chang, Antoine Venault

**Affiliations:** R&D Center for Membrane Technology, Department of Chemical Engineering, Chung Yuan Christian University, Chung-Li 32023, Taiwan

**Keywords:** phase-inversion, antifouling materials, blending, pressure-driven membranes

## Abstract

Membrane technology is an essential tool for water treatment and biomedical applications. Despite their extensive use in these fields, polymeric-based membranes still face several challenges, including instability, low mechanical strength, and propensity to fouling. The latter point has attracted the attention of numerous teams worldwide developing antifouling materials for membranes and interfaces. A convenient method to prepare antifouling membranes is via physical blending (or simply blending), which is a one-step method that consists of mixing the main matrix polymer and the antifouling material prior to casting and film formation by a phase inversion process. This review focuses on the recent development (past 10 years) of antifouling membranes via this method and uses different phase-inversion processes including liquid-induced phase separation, vapor induced phase separation, and thermally induced phase separation. Antifouling materials used in these recent studies including polymers, metals, ceramics, and carbon-based and porous nanomaterials are also surveyed. Furthermore, the assessment of antifouling properties and performances are extensively summarized. Finally, we conclude this review with a list of technical and scientific challenges that still need to be overcome to improve the functional properties and widen the range of applications of antifouling membranes prepared by blending modification.

## 1. Introduction

### 1.1. Membrane Technology

The global water shortage has resulted from population growth and industrialization and is a key challenge that humanity faces in the 21st century. Continuous progress on wastewater treatment technologies has been made to improve water reuse and reduce water shortages. Among them, the development of thin-layer membranes, working as selective agents between two phases and regulating the permeability of substances, has made tremendous progress.

Pressure-driven membranes are commonly categorized into four groups depending on their pore size and the type of filtration involved: microfiltration (MF), ultrafiltration (UF), nanofiltration (NF), and reverse osmosis (RO) membranes [1,2,3,4]. Owing to their ease of fabrication and operation, high selectivity rates, energy savings, and high-level modularity, membranes have made significant contributions to address the challenges associated with resource and energy scarcity and have been increasingly used in applications such as desalination, water treatment, and food and pharmaceutical manufacturing [5,6,7]. Myriads of membranes used in wastewater treatment are formed from either organic or inorganic membranes [8,9]. The prior is predominantly based on a variety of polymers such as polyamide (PA), poly(vinylidene fluoride) (PVDF), polysulfone (PS), polyethersulfone (PES), polypropylene (PP), polyimide (PI), poly(ether imide) (PEI), poly(tetrafluoroethylene) (PTFE), polycarbonate (PC), polyetherketone (PEEK), polypiperazine (PPZ), polyacrylonitrile (PAN), cellulose acetate (CA), and so on. Polymeric membranes offer facile and straightforward fabrication methods for a wide range of pore sizes, and simple modification techniques that allow for cheap and easy industry scalability [10,11]. However, these materials are prone to fouling and susceptible to extreme operating conditions such as pH, temperature, pressure, etc. On the other hand, inorganic membranes are tailored from ceramics or metals which are characterized by having stronger resistance against harsh operating conditions, although their widespread applications are greatly hampered by the difficulty of their processability, and fabrication techniques, and expensive cost [12]. Furthermore, inorganic membranes are mostly limited to MF and UF applications to date [9]. Hence, the polymeric membrane is still considered the primary choice for the vast majority of the studies on membrane technology.

### 1.2. Preparation of Porous Membranes via the Phase-Inversion Processes

**Figure 1:**
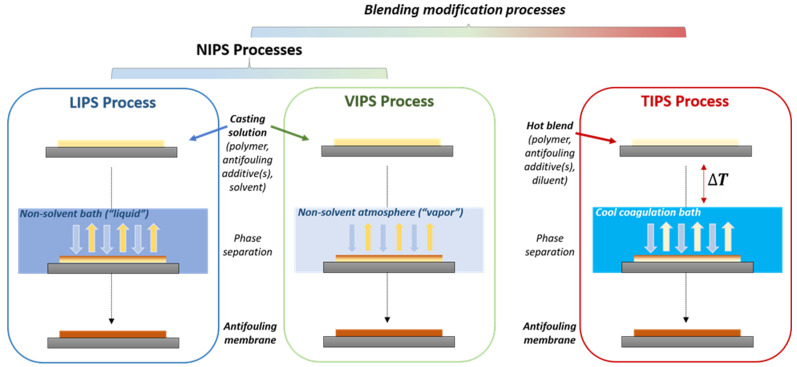
Schematic of phase-inversion processes for the formation of flat-sheet antifouling porous membranes via blending modification.

Phase-inversion processes are well-established techniques for fabricating porous polymeric membranes. In general, phase-inversion is a demixing process that converts a homogeneous polymer casting liquid solution to a solid film in a controlled manner (Figure 1) [13]. The final morphology of the membranes can be determined by the demixing process which is greatly influenced by the thermodynamics, viscoelasticity of the casting solution, and kinetics of the solvent/non-solvent upon precipitation.

Phase-inversion processes can be induced by the utilization of a non-solvent (Non-solvent Induced Phase Separation, NIPS) which can be liquid (wet-immersion or Liquid-Induced Phase Separation, LIPS) or vapor (Vapor-Induced Phase Separation, VIPS). It can also be triggered by a rapid change in temperature (Temperature-Induced Phase Separation process, TIPS).

In LIPS, after casting a homogeneous solution on an adequate substrate, it is immersed in a non-solvent coagulation bath for a desired time. As a result of the exchange of the solvent and the non-solvent, precipitation occurs, and a membrane film is produced. The excess of solvent is then removed by extensive washing of the membrane in water [14,15,16]. UF membranes are usually prepared by this method. In the VIPS process which leads to MF membranes, the homogeneous solution is exposed to vapors of the non-solvent under controlled conditions of humidity, temperature, and time. It is sometimes combined with LIPS to control the morphology of the top surface and increase its the porosity [17]. In TIPS, a rapid change in temperature of the polymeric system caused by immersion of a hot polymer/diluent system in a cold/cool immersion bath induces phase-inversion. In NIPS processes (LIPS and VIPS), the nature of the solvent/non-solvent system and the concentration and composition of the polymer solution are key aspects that impact the phase-inversion approach for membrane production [1,18,19]. In VIPS, one has to consider the impact of the process parameters (example: relative humidity, exposure time to vapors). This process has been extensively reviewed recently and one is invited to refer to the study of Ismail et al. for relevant information [20]. As for the TIPS process, key parameters to consider are the temperatures of the dope and of the coagulation bath, and, if hollow fibers are produced, the dope flow rate and the air gap between the spinneret and the coagulation bath [21,22,23,24].

Phase-inversion methods require relatively simple equipment and can be readily implemented. DI water is frequently used as a non-solvent medium (in LIPS, VIPS) or a coagulation medium (in TIPS), which makes these processes relatively cost-effective. Furthermore, they are applicable to a large variety of materials. The major current drawback is the toxicity of the solvents used. The addition of organic/inorganic additives for antifouling purpose may also cause solubility issues, resulting in the production of inhomogeneous dispersions in the matrix membrane which in turn diminishes the stability of the membranes and ultimately their antifouling performances.

### 1.3. Membrane Fouling and Fouling Mitigation Techniques

Fouling, the “Achilles’ heel” of membrane technology, is a pervasive issue that if not prevented or acted upon, could leave many water treatments in futility. Membrane fouling is caused by the deposition or adsorption of pollutants or foulants on the surface of the membranes and/or penetration into the pores [25], which then leads to a series of operational and performance issues such as flux decline, decreased permeate purity, and overall poor separation performance. In general, foulants are categorized into inorganic foulants, organic foulants, biofoulants, and composite foulants. Inorganic foulants include inorganic scales, colloidal inorganic substances, etc. Organic foulants encompass oils, organic solvents, organic dyes, natural organic matter (NOM), and so on. Biofoulants, on the other hand, consist of one or more types of biomolecules or cells. Lastly, composite foulants are the combination of the inorganic, organic and biofouling foulants [26]. Although inorganic scaling/fouling poses serious threat to membrane performance, it is inhibited through pre-treatment of the feed or optimization of operating conditions [25]. Therefore, studies on the development of antifouling membranes are geared towards mitigation of organic foulants and biofoulants. Jiang and colleagues published in 2015 an interesting review that tackled the fouling mechanisms of various foulants [25], which helped inspire various antifouling studies.

Proteins, commonly used biofoulants for many studies, exist as amphiphilic compounds due to the existence of both hydrophilic polar groups and hydrophobic non-polar groups. Protein fouling on the membrane surface is generated by protein–membrane interactions prompted by hydrophobic interaction by the protein and membrane. In an aqueous environment, the hydrophilic groups of the proteins can bind with the water molecules loosely adhered on the surface of the membrane via hydrogen bonding and electrostatic interactions. These loosely adhered water molecules could be easily displaced, increasing the chances of the hydrophobic portions of the proteins to form hydrophobic–hydrophobic interactions with the membrane. In addition, if the pH of the solution is equal to the iso-electric point of the proteins, it becomes strongly hydrophobic, in turn facilitating the hydrophobic interactions of protein–membrane. Moreover, proteins tend to aggregate at their iso-electric point, enhancing the protein–protein hydrophobic interaction. Hence, this cascades into enhanced fouling as the aggregated proteins form a hydrophobic interaction with the initially adhered proteins on the membrane surface [26,27].

Natural organic matter (NOM) such as humic acid (HA) and sodium alginate (SA) consist of hydrophobic aliphatic and aromatic moieties, allowing these types of foulants to be adsorbed on the membrane surface through hydrophobic interactions. Organic dyes (e.g., Congo red, methyl blue, rhodamine B, malachite green, methyl orange, rose Bengal dye, acid black 210, and so on [28,29,30,31]) are interesting foulants due to their complex structure, and electrical charge. In addition, dye effluents have high salinity due to the addition of salts (common practice by textile industries to enhance dye uptake) [28,32], which makes the fouling mechanism by organic dyes complex. Distinct from proteins, dye molecules aggregate through ionic association, hydrophobic interaction, hydrogen bonding to form hydrated ions and spherical or flake aggregates in an aqueous solution. Moreover, mutual association and aggregation between adjacent dye molecules on the membrane surface could be induced through hydrophobic interactions. Due to the charged nature of the dye molecules, it can induce concentration polarization, affecting the flux. Additionally, the adsorbed dye can form strong chemical bonds with the groups on the membranes surface (or within the membrane pores) [29]. Oil foulants can exist as free oil, emulsified oil and/or dissolved oil which could form fouling on the membrane surface through hydrophobic–hydrophobic interactions. As a result of the high tendency of the oil droplets to coalesce, this can form an oil layer on the surface of the membrane which makes the penetration of water in the succeeding cycles more challenging. Also, in surfactant stabilized emulsifiers, the surfactants used are amphiphilic which could result in penetration and clogging of the membrane pores [12].

Hence, polymeric membranes must be conferred with antifouling properties that are tailored to alleviate various interactions between the foulant and the membrane surface, and the interaction that transpires between the foulant and adsorbed foulant on the membrane surface. These could be achieved through increasing surface hydrophilicity, enhancing the surface charge, and decreasing the surface roughness of the membranes [33].

### 1.4. Modification Techniques for Antifouling

**Figure 2:**
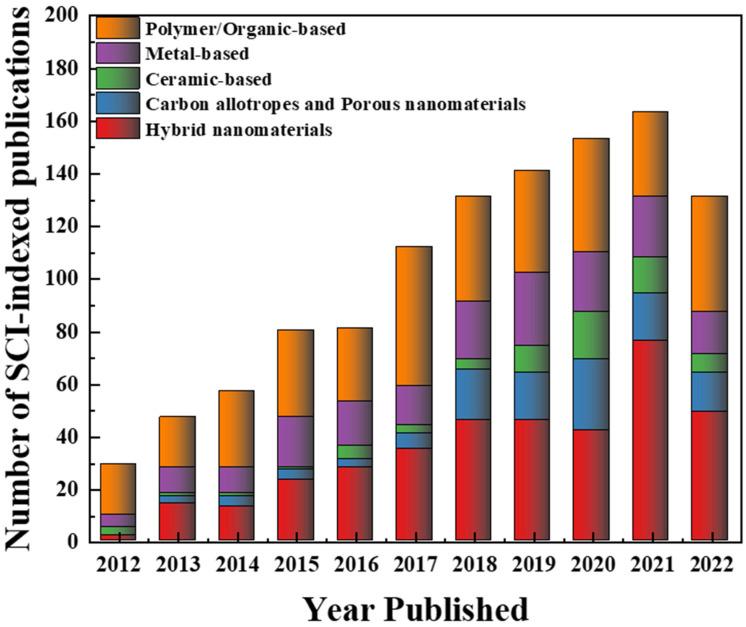
Literature survey of antifouling membranes prepared via blending modification from 2012–2022 obtained from the Web of Science.

**Figure 3:**
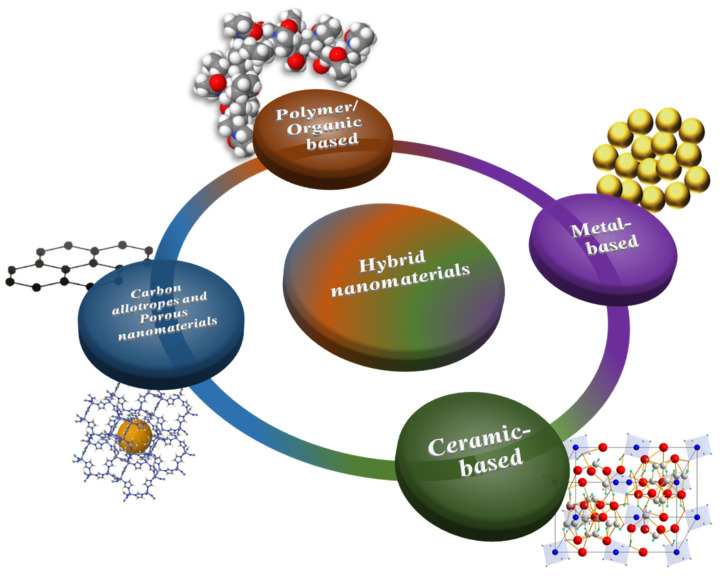
Schematic illustration of the different groups of additives used to modify polymeric membranes by blending.

In order to mitigate fouling and maintain high permeability/separation performance, porous membranes can be modified via several techniques including grafting polymerization, layer-by-layer deposition, dip-coating, spray coating, spin coating, etc. [5,19,34,35,36]. These techniques modify the top surface of the membrane and involve a post-treatment step [37]. On the other hand, physical blending or simply “blending” involve the process of incorporating the matrix polymer with inorganic nanofillers or hydrophilic and/or amphiphilic polymers in the casting solution before applying a phase-inversion process (LIPS, VIPS or TIPS). This modification technique is a one-step process since the membrane is formed/modified all at once. Many recent reports have shown the suitability of this technique to improve the hydrophilic properties of membranes, resulting in fouling mitigation [34,38,39,40]. Researchers have worked on the modification of polymeric membranes using various additives including polymer-based materials such hydrophilic or amphiphilic polymers, biopolymers and nature-derived materials [41,42,43,44,45,46], metal-based nanomaterials [47,48,49,50,51,52,53,54,55,56,57,58], ceramic-based nanomaterials [59,60,61,62,63,64,65,66], carbon allotropes (graphene-based materials, carbon nanotubes, etc.), layered and porous nanomaterials (e.g., zeolites, metal-organic frameworks (MOFs), etc.) [67,68,69,70,71,72,73], and hybrid nanomaterials (which includes the combination of two or more materials) [74,75,76,77,78,79] in order to develop the hydrophilic characteristics and antifouling capability of membranes necessary for water treatment or their application in the biomedical field. Distinct from other methods, blending modification does not require any additional steps during the fabrication of polymeric membranes or mixed matrix membranes (MMMs), making it a relatively simple and fast method; hence, considerable attention has been given to this over the past decade as illustrated by the number of research publications in Figure 2. A schematic diagram representing each material groups are shown in Figure 3.

### 1.5. Objectives of the Article

This review article explores the published recent advances on the theme of antifouling membranes focused on one-step formation/modification of membranes or blending modification by phase-inversion. As a review on biofouling of water treatment membranes was published in this journal 10 years ago [80], we tried to solely focus on the period 2012–2022. We begin with exploring the antifouling materials belonging to the class of polymers/organic, metals, ceramics, carbon allotropes and porous nanomaterials, and hybrid nanomaterials. Then, the effects of antifouling materials on membrane morphology are investigated. In addition, the methods used to assess the antifouling properties of membranes are discussed, as well as the performances of the modified membranes, in comparison with the pristine membranes. Finally, future perspectives are proposed.

## 2. Antifouling Materials for Polymeric Membranes

The key problem in wastewater treatment utilizing polymer-based membranes is developing low-cost and high-performance antifouling membranes. Membrane fouling lowers the lifespan of membranes [1,81,82,83]. Real-world membrane use requires the creation of cost-effective wettable polymeric membranes. The choice of membrane composition and the control of morphological features both have a substantial effect on antifouling properties as they impact the membrane wettability [63,83].

When weak interactions between the foulant and the membrane take place, reversible fouling occurs, which may be readily eliminated by a physical cleaning of the membrane. However, strong interactions between the foulant and the membrane surface arise in irreversible fouling, resulting in the production of a persistent fouling layer that cannot be cleaned by ordinary procedures. Biofouling is often regarded as irreversible. For example, bacteria that stick onto the surface of the membrane can grow and reproduce, eventually covering the whole membrane surface. How to enhance the membrane’s antifouling capability by reducing interactions between the material and bacteria/cells has become a crucial topic in membranes applied to water treatment and in the biomedical field and requires the use of antifouling materials. The high degree of surface hydrophilicity imparted by a suitable surface chemistry result in efficient fouling mitigation in aqueous media [1,7,15,84,85]. Research teams worldwide have investigated the blending of polymers traditionally used for membrane fabrication with various types of antifouling materials belonging to different classes. What follows is a presentation of these additives, which are categorized into five types according to their intrinsic characteristics: polymer/organic-based, metal-based, ceramic-based, carbon allotropes and porous nanomaterials, and hybrid nanomaterials, as represented in Figure 3. We also present a discussion of their effect on the performance of the membranes.

### 2.1. Polymer/Organic-Based Additives

The majority of polymer materials used for the large-scale production of porous membranes applied in wastewater treatment are hydrophobic. So, the use of hydrophilic or amphiphilic polymers has been investigated. Recent works published in the last 10 years starring these polymeric materials are summarized in Table 1 and introduced as follows. It is important to note that hydrophilic polymers such as poly(ethylene glycol) or PEG, and poly(vinylpyrrolidone) or PVP are commonly used as porogens or pore formers; in this review, we investigated studies that utilized these polymers as antifouling modifiers not as porogens.

#### 2.1.1. Poly(ethylene glycol) and Its Derivatives

Poly(ethylene glycol) (PEG) exists in a variety of molecular weights (Mws), for example PEG 200, PEG 400, PEG 6000 or PEG 20,000 where the number following “PEG” refers to the average molecular mass. Ma et al. studied the influence of PEG Mw and content on the properties of polysulfone (PSf) membranes [42]. Increasing the PEG Mw significantly enhanced the pure water flux (PWF) while decreasing the rejection performances, as it affected the pore size. From there, they selected PEG400 and showed that increasing its content led to higher permeability still maintaining bovine serum albumin (BSA) rejection, which could be explained by the presence of more pores of similar size, that is, by the pore-forming effect of PEG. Note though that boosting the PEG content also reduced the mechanical properties of the membranes due to the increased porosity. Thus, not only the molecular weight but also the PEG content had to be optimized.

As a result of its hydrophilic nature, PEG may easily leach away during membrane washing or even during filtration, resulting in reduced hydrophilicity and antifouling performance [1,87,89]. So, the emphasis has been put in the past decade on the design of amphiphilic additives composed of both hydrophobic and hydrophilic units. The hydrophobic units ensure stability of the antifouling material in the membrane system, and so, they are permitted to maintain for a longer time period the antifouling property [88]. The hydrophilic segment tends to be oriented towards the membrane surface/pore surface during phase-inversion which enables the increase in the membrane hydrophilicity and its antifouling ability. Copolymers of PEG, poly(ethylene glycol) methyl ether (PEGME), and poly(ethylene glycol) methyl ether methacrylate (PEGMA) have been reported [41,86,88,90,92,93,153,154]. Differing from PEG, PEGME and PEGMA derivatives have a -CH_3_ terminal which decreases the chance of leaching out [154]. However, this cannot be guaranteed over a long period of time. Hence, strategies have been developed in order to resolve this issue. In some cases, the hydrophilic segment was grafted to the matrix polymer, and the resulting copolymer blended with the pristine polymer. A major advantage of this technique is the compatibility between the materials and the strength of the hydrophobic interactions established between the copolymer and the membrane main polymer. For instance, Wu et al. first grafted PEGMA to PVDF, and then blended the grafted copolymer with pristine PVDF [92,93]. Similarly, PEGMA was grafted to PVC and the resulting material blended with PVC to form membranes by LIPS [91]. The use of copolymers requires an optimization step in order to maximize stability and antifouling. Too many hydrophobic segments lead to high stability but poor antifouling property. Conversely, too many hydrophilic segments lead to excellent but not sustainable fouling mitigation. Gao et al. introduced an amphiphilic random comb copolymer containing PEG and poly(dimethylsiloxane) (PDMS) which blended with PES. PEG-*r*-PDMS was prepared using the free radical polymerization process [88]. They observed that the increased amount of PDMS in the amphiphilic copolymer PEG-*r*-PDMS resulted in higher WCA, but stability was achieved. In our previous work, we investigated the effects of tailoring both the composition and molecular weights of PS-*r*-PEGMA copolymers [90], and we found that longer chains led to better antifouling properties, not because of a higher overall hydration level, but because of a tighter hydration layer.

#### 2.1.2. Poly(vinylpyrrolidone)

PVP has similar properties to PEG, as both are hydrophilic in nature and used as pore-forming agents in polymeric membranes. Although there are stability concerns, PVP still remains an important material to tune the surface properties of membranes prepared by blending modification. For instance, it was recently used by Son et al. to tune the permeation and antifouling properties of PES membranes [43]. They specifically laid the focus on high PVP concentrations but concluded that large additive amounts (15 wt%, 20 wt% in the casting solution) were not desired as they induced the formation of a dense layer, decreasing water permeability. Instead, 10 wt% PVP in the casting solution seemed to lead to the best combination of membrane permeability, rejection, and antifouling property. Kanagaraj et al. investigated the formation of polyetherimide (PEI) with various concentrations of PVP (0–8 wt%) and reported that the larger concentration permitted to maximize the PWF (147.1 L/m^2^ h) and minimize protein adsorption [94]. However, it was at the expense of the membrane’s tensile strength and of protein rejection due to the pore forming effect of PVP in this concentration range. Similar to PEG, PVP is available over a large range of molecular weights. For a given additive content, it is clear that the choice of a larger Mw will result in larger casting solution viscosity, which should help in decreasing mass transfer rates during phase-inversion and lock the additive in the main matrix polymer. Vatsha et al. [96] investigated the effect of using high concentrations of PVP 40 k as modifier for PES. They reported that at 10 wt% of PVP (for 16 wt% and 18 wt% PES), significantly increased the hydrophilicity, but induced the formation of dense surface structure. These reports show that by using larger amounts of PVP, denser structures are obtained, as the total volume fraction of solid content increases. It suggests that PVP can still be retained in the matrix polymer and can be of interest to increase the surface free energy of the membrane through both a change in surface chemistry and surface structure (less pores), which would benefit fouling mitigation, although it may reduce the membrane permeability. Similarly, Vatsha et al. used similar logic, and finally, PVP has been recently combined with polydiol terephthalate to form an amphiphilic triblock copolymer, that resulted in the formation of stable antifouling PSf membranes [95].

#### 2.1.3. Cellulose Nanocrystals

Cellulose nanocrystals (CNC) additives are biocompatible, renewable, environmentally friendly, and they exhibit excellent mechanical strength. Due to their numerous hydroxyl groups and unique mechanical strength, CNC additives have received increasing attention to improve the surface hydrophilicity, permeability, antifouling properties, and mechanical strength of blended membranes [99,100,101]. The presence of hydroxyl groups facilitates the formation of hydrogen-bonding networks, hence the hydration of films. Zhang et al. incorporated CNC into PES UF membranes [102]. They blended different amounts of CNC (0.1–5.0 wt%). The virgin PES membrane displayed a WCA of 66°, which dropped to 43° for the membrane containing 5.0 wt% CNC. Meanwhile, BSA rejection was increased from 93% (virgin membrane) to 97% due to the formation of smaller pores, and the flux recovery ration (*FRR*) enhanced (from 51 to 90%). Nevertheless, the blended membranes’ tensile strength and elongation at break both decreased dramatically with the CNC loading, which may result from heterogeneous dispersion of the nanocrystals into the polymeric matrix. Similarly, Zhou et al. reported that the agglomeration of the nanocrystals could occur past a certain loading, leading to the formation of “holes” (large pores) seen on the membrane surface, responsible for a decrease in the mechanical properties [101]. Lv et al. explored the incorporation of CNC into PVDF membranes and reported the significant effect of the CNC loading on the membrane structure [98]. While low loadings (0.7–1.4 wt%) were associated with homogeneous dispersion of the CNC and denser surfaces, higher loadings (1.4–2.8 wt%) seemed to arise in the formation of more surface pores, until large defects were observed for the highest loading (4.2 wt%). Similar reports [102,103] suggest the need for adjusting the CNC content in order to take full advantage of its effect on both surface hydrophilicity and mechanical reinforcement of the matrix. Clearly, agglomeration readily occurs past a concentration threshold, which can severely decrease the membrane mechanical strength while homogeneous dispersion reinforces the structure, while contributing to the mitigation of fouling.

Interestingly, the porosity and pore size reported in these papers have shown to increase with increasing CNC concentrations. It is true that agglomeration of CNC is likely possible, which leads to poor dispersion in the polymeric matrix (especially for hydrophobic polymer such as PVDF), which could then result in decreased surface and bulk porosity and pore size of the prepared membranes, as the total solid content of the casting solution increases. Yet, the opposite has been observed in previous works for CNC. In addition, the substructures of the prepared membranes from these studies (both pristine and modified) display large macrovoids extending throughout the entire cross-section, which from former knowledge indicate weak points in membranes [155]. Although it can be argued that the highly hydrophilic nature of CNC could promote faster demixing rates subsequently forming large macrovoids, however, these claims should be substantially backed by kinetics data alongside thermodynamics and viscosity analyses.

The hydrophilic functional groups of CNC endow polymeric membranes such as PVDF, PES, CA, etc. with enhanced hydrophilicity that helped to mitigate the fouling; however, more work is needed to better understand the effects of CNC on the morphology of polymeric membranes, and how it could affect the long-term antifouling stability of CNC blending modified membranes.

#### 2.1.4. Poly(vinyl alcohol)

PVA is an extremely hydrophilic polymer prepared by partial/full hydrolysis of poly(vinyl acetate). It exhibits outstanding thermal, chemical, and mechanical properties. However, its properties strongly depend on the degree of hydroxylation [105]. Nevertheless, it can be solubilized in a quite broad range of solvents, making it suitable for the blending modification of numerous types of polymer membranes [104,105,106], although compatibility issues arise with highly hydrophobic polymers [107]. Yuan et al. recently modified a PES membrane by blending different weight ratios (0–30 wt%) of PVA [106]. They reported morphological changes as well as an increase in membrane hydrophilicity. The authors showed that increasing the amounts of PVA in the casting solution increased the PWF (from 15 to 131 L/m^2^ h) while decreasing the BSA rejection (from 81% to 61%) due to the formation of macrovoids in the sublayer and the increased number of pores on the surface. Moreover, the *FRR* could be increased from 52% to 92.6%, indicating the excellent antifouling capabilities of the additive. Zhang et al. fabricated PVDF/PVA membrane [107], working with relatively low additive content (<0.5 wt%) because of the poor compatibility of PVDF and PVA. In spite of that, by fine-tuning the PVA content in the casting solution (0.1 wt% PVA), they managed to prepare stable membranes with improved antifouling properties.

#### 2.1.5. Poly(acrylic acid)

Poly(acrylic acid) (PAA) enables a significant increase in water trapping in a membrane and is characterized by its biocompatibility. Furthermore, it is an effective crosslinking agent; its large number of carboxyl groups allows for easy adsorption or chelation of heavy metal ions, so it has been used for their removal [108,109]. Although the impact of PAA on the hydrophilicity and antifouling property of blending modified membranes can be demonstrated, the stability of the modifying agent in the matrix membrane can also be an issue. Due to its water solubility, PAA elution is inevitable when mixed directly with another polymer. Similarly to PVP, increasing the molecular weight permits one to address this issue, as it improves the tangling of PAA chains with the main polymer matrix chains. Ounifi et al. examined the effect of PAA on the removal of cadmium by cellulose acetate (CA) membranes [108]. The blending of 15 wt% of PAA into the CA matrix membrane led to a sharp decrease in the WCA from 71.5° to 25.0°, while the porosity increased from 44.6% to 75.6%. The addition of PAA facilitated water diffusion through the membrane by improving hydrogen bonding interactions. Thus, improved solvent/non-solvent exchange rates during membrane formation resulted in the formation of finger-like structures, and, in turn, a larger membrane porosity. The decrease in WCA was readily reasoned by the presence of numerous carboxylic groups. As a result, the modified CA/PAA membranes outperformed the convectional CA membrane in terms of water permeability. Interestingly, Cd rejection and humic acid (HA) rejection (99.9%) were also improved. In summary, PAA not only permits the mitigation of fouling of blending modified membranes, but also the removal heavy metals from water bodies, which is undeniably essential to safe wastewater reuse.

#### 2.1.6. Polydopamine

Polydopamine or PDA is a product of dopamine polymerization using oxidizing agents such tris (tris hydroxymethyl aminomethane), ammonium persulfate, sodium periodate or sodium chlorate, copper sulfate buffer solution, and H_2_O_2_ [111,156]. The abundance of polar hydrophilic groups in PDA makes it a highly sought-after hydrophilic modifier for antifouling membranes. However, its high affinity towards water makes it highly vulnerable to leaching, allowing for the formation of large pores and increased porosity of the modified membrane as described in the work of Ang et al. [112].

Aside from endowing hydrophilic properties to the polymeric membranes, PDA can be easily functionalized with different functional groups to modify the surface charge of the membrane. Recently, Kallem et al. [114] functionalized PDA with –SO_3_H and subsequently incorporated into the PES matrix. Sulfonated PDA enhanced the surface negative charge of PES, increasing the electrostatic repulsion forces between the foulants (such as SA, HA and BSA) and the membranes’ surface. NF PES membrane exhibited increased rejection towards mono and divalent salts through the incorporation of quaternized PDA due to the increased presence of positively charged quaternary ammonium functional groups (-N^+^(CH_3_)_3_) [110]. Alongside being a stand-alone hydrophilic additive, PDA can also be used to functionalize nanomaterials such GO [157], nanoclays [158], metal nanoparticles [159], and so on.

#### 2.1.7. Amphiphilic Copolymers

As mentioned in the previous section of the review, organic fouling and biofouling could be mediated by hydrophobic–hydrophobic interactions (among others prefaced in Section 1.3) with the membranes’ surface. Logic dictates that blending hydrophilic additives could confer conventional polymeric membranes with antifouling properties. However, these additives are highly soluble in water hence, they leach out in the coagulation bath and during membrane operation, ultimately causing the membranes to suffer from fouling. In order to keep the additives in the membrane matrix and enhance the stability of the blending modification, developments of amphiphilic copolymers containing both hydrophobic and hydrophilic segments were explored [95,125,126,127,128,129,130]. The hydrophobic segments act as anchoring fragments as they form hydrophobic–hydrophobic interactions with the polymeric matrix, thus preventing the additive from leaching out. Meanwhile, the hydrophilic segments endow the membrane with increased surface hydrophilicity as a result of surface aggregation during phase-inversion process. In this, the hydrophilic segments migrate towards the surface and promotes the formation of hydration layer through hydrogen bonding or ionic solvation which prevents the foulants from establishing hydrophobic–hydrophobic interactions with the membranes’ surface [25,160]. With higher concentrations of amphiphilic copolymers, surface segregation becomes more apparent, and the surface roughness of the prepared sample is increased. In alleviating foulant adsorption on the membranes’ surface, it was found that smoother surfaces are able to better repel foulants as they have better steric repulsion. On the other hand, rough surfaces could induce decreased steric repulsion as valleys and groves on the surface of the membranes make the polymer brushes easy to be compressed, which decreases the distance between the foulant and membranes’ surface. This phenomenon leads to a decrease in the Gibbs free energy of the system, and as it continues to decrease, fouling becomes more thermodynamically possible [25,161]. Hence, it is significant to find the balance between having strong hydration layer and good steric repulsion.

In utilizing amphiphilic copolymers, several parameters should be considered: (1) choosing between random and block copolymers; (2) regulating the molar ratios of the hydrophobic and hydrophilic segments; and (3) adjusting the chain lengths of the hydrophobic and hydrophilic fragments. The synthesis of random copolymers requires fewer complex procedures than block copolymers, hence, it can be easily scaled-up. Although random copolymers have wider molecular weight distribution, resulting in less stable assemblies compared to block copolymers, [162] random copolymers undeniably still offer good antifouling properties as exhibited from previous works on PS-*r*-PEGMA [90,163] PDMS-*r*-PEG random copolymers [128]. Despite having random assembly, 85–90% of PS-*r*-PEGMA remained in the PVDF matrix after 24 h immersion at 37 °C with constant stirring (simulating bacteria attachment tests) [90], while around 85–90% of PDMS-*r*-PEG remained in PVDF after 30-day immersion at 30 °C [128]. Of course, it is undeniable that block copolymers have better long-term antifouling stability and reusability as exhibited in previous works [95,125,126,127,160,164,165]. Zhang and colleagues reported that after long-term filtration, Pluronic F127/PMIA membrane water flux almost remain unchanged [160]. In choosing between random and block copolymers, we need to take into consideration the complexity of the copolymer synthesis, and long-term applications of the prepared membranes. Although random copolymers can be easily synthesized, their potential can be offset by their stability in the membrane matrix for long-term filtration and harsher conditions. On the other hand, block copolymers are ideal for long-term filtration applications. However, it requires complex synthesis which are costly to be scaled-up for industry.

Regulating the molar ratios of the hydrophobic and hydrophilic segments of the amphiphilic copolymers is also a significant aspect that needs to be considered to ensure effective antifouling properties and good stability in the membrane matrix. We recently investigated the effect of changing the hydrophobic/hydrophilic in PS-*r*-PEGMA [90]. Increasing the hydrophilic segments provided better hydration capacity, however, the stability of the copolymer in the matrix was significantly decreased due to the fewer “anchoring” segments that induces hydrophobic–hydrophobic interactions with the membrane matrix. Additionally, when only PEGMA was added (without polystyrene units), the contact angle of the prepared membrane remained high (~100°) which could be due to the inhomogeneous immobilization of hydrophilic segments in the membrane matrix. On the other hand, when polystyrene moieties are greater than PEGMA, the antifouling ability of the modified membrane was compromised, which led to the increased adsorption of protein on the surface. Aside from this, fine-tuning the chain lengths of the copolymer needs to be considered. Previous reports show that the strength of the hydration layer is highly correlated with chain length as more “water-hydrophilic units” interactions are formed through electrostatic interactions and hydrogen bonding. The water molecules attached to the surface could be categorized as free water, freezable water, and non-freezable water. Since free water is the farthest from the surface, it has the weakest bonding thus, it is very mobile and could be easily displaced [166]. Meanwhile, freezable water has moderate mobility, and could not be as easily displaced as free water. On the other hand, due to the proximity of the non-freezable water to the surface, it forms the strongest interaction to the surface, making it the hardest to remove. It has been investigated that with longer chains, more non-freezable water is formed, strengthening the hydration layer, and providing increased antifouling properties [90,167].

#### 2.1.8. Zwitterionic Additives

Zwitterionic materials have both large dipole moments and charged groups. This results in the formation of a stronger hydration layer, compared to other types of non-charged antifouling moieties, essential to long-term fouling mitigation. These materials are endowed with the same number of cations and anions on their polymer chains, found on the same monomer units. Quaternized ammonium is a common cation, and common zwitterionic moieties are categorized as sulfobetaine (SB), carboxybetaine (CB), or phosphorylcholine (PC) based on the nature of their anions (sulfonates in SB, carboxylates anions in CB and phosphonates anions in PC). As a result of their propensity to associate with a large number of water molecules via hydrogen bonding and electrostatic interactions, zwitterionic materials are extremely hydrophilic, and their overall set of properties make them ideal antifouling materials [115,121,168,169,170]. However, and distinct from the other materials above-mentioned, zwitterionic units need to be combined with hydrophobic segments in order to prepare blending modified membranes. If not, the large polarity difference between the zwitterionic material and the membrane material makes the formation of a homogeneous casting solution extremely challenging if not impossible.

Li et al. developed an amphiphilic zwitterionic copolymer by grafting poly(sulfobetaine methacrylate) (PSBMA) moieties on PVDF chains, and then blended the resulting PVDF-*g*-PSBMA material with PVDF to form membranes by phase-inversion [118]. As for PVDF-*g*-PEGMA (Section 2.1.1), grafting the hydrophilic monomer on the same polymer used in the membrane preparation greatly enhances compatibility and stability of the system. When the PVDF-*g*-PSBMA content in the casting solution was increased to 14 wt%, the PWF was drastically enhanced from 121.9 L/m^2^ h (virgin membrane) to 239.1 L/m^2^ h. Moreover, compared to the virgin PVDF, the blended membranes showed a lower flux reduction ratio during filtration of a protein solution. The *FRR* for the virgin and of the best membrane were 51.5% and 81.2%, respectively, proving the efficiency of the zwitterionic material to mitigate fouling. Zhao et al. designed sulfonated polyaniline (SPANI) [126]. This material is self-doped with both positive and negative charges, resulting in the formation of a zwitterionic material. The blending modification of PVDF membranes with SPANI highly improved their hydrophilicity (WCA dropped from 92.0° to 29.0°) and consequently, their PWF (which increased from 97 to 160 L/m^2^ h). As a result of the excellent ion hydration, the absorption of contaminants was prevented. Our group modified PVDF membrane with a random zwitterionic copolymer containing styrene, sulfobetaine methacrylate, and ethylene glycol methacrylate units (PS-*r*-PEGMA-*r*-PSBMA) [117], and we also changed styrene to methyl methacrylate units [116]. The key aspect of this set of studies was the use of PEGMA as a solubility-enhancing unit, facilitating the blending of the zwitterionic material with PVDF. The zwitterionic MF membranes prepared by the VIPS process were shown to resist fouling caused by a large variety of biofoulants including blood components. The design of blood compatible membranes by blending modification was also investigated, using a copolymer of 2-methacryloyloxyethyl phosphorylcholine and methacryloyloxyethyl butylurethane (MPC-derivative), hence containing PC units popular for their hemocompatibility [122]. Finally, Maggay et al. recently investigated the effect of a zwitterionic copolymer derived from 4-vinylpyrridine (denoted as zP(S-*r*-4VP)) on the antifouling properties of PVDF membranes prepared by VIPS [119] and of PSf membranes formed by a dual-bath process [120]. Interestingly, this material permits the preservation of the antifouling properties of the membranes even after exposure to hot steam (steam sterilization process). This was also a key feature of a recent study staring a copolymer containing sulfobetaine methacrylamide functions (SBAA) [123]. While SBMA can be hydrolyzed, it appears that SBAA is more stable when exposed to hot steam, making its derivatives potential candidates for the formation of antifouling membranes for biomedical applications.

#### 2.1.9. Nature Derived Biopolymers

Growing environmental concerns have prompted many researchers to delve into nature-derived hydrophilic polymers (listed in Table 1) due to their easy access, reasonable cost, non-toxicity, and intrinsic hydrophilicity [144,171]. These biopolymers have been well-documented to improve the hydrophilicity of polymeric membranes, which could help enhance the flux whilst mitigating foulants by forming hydration layer. Although these biopolymers offer promising antifouling properties with less environmental risks, their widespread applications are still being challenged by their solubility in organic solvents, stability in the membrane matrix, and scalability of their synthesis procedures (extraction of the desired compound from natural raw materials). For example, chitosan or its derivatives are insoluble in common solvents [137,139], which makes the modification of the bulk of the membrane difficult. Moreover, since these biopolymers have many organic linkers [131,142,144] that imparts them with hydrophilic properties and polarity, these biopolymer additives could be easily washed off during immersion in water. Although this is beneficial in promoting a porous structure during phase-inversion, prolonged immersion in water such as in water treatment operation could cause these biopolymers to leach out over time and could eventually lead to decreased antifouling properties. Additionally, since they are derived from nature, the steps needed to achieve the “purified state” of the biopolymer are no easy feat. Moreover, the yield from these procedures should also be taken into consideration. Despite being considered as more sustainable alternative additives, there are still a lot of factors that limit the potential of biopolymers from being fully realized in membrane technology.

#### 2.1.10. Concluding Remarks on the Use of Polymeric Additives

Hydrophilic (both synthetic or natural) or amphiphilic polymers and copolymers have remained popular in the past 10 years to improve the antifouling properties of membranes formed in one step by phase-inversion. Their incorporation to the membrane system does not just result in better mitigation of fouling, but also in drastic changes in membrane structure and arising properties (pore size, porosity) through the effect of the additive on membrane formation mechanisms. The maximum concentration of the antifouling additive has to be carefully adjusted in order to reach the desired fouling mitigation while maintaining the mechanical properties. This aspect has to be highlighted as the LIPS process applied to casting solutions containing hydrophilic/amphiphilic additives often leads to membranes with larger macrovoids than those seen in the pristine matrix. These macrovoids surely facilitate transport during filtration (provided also that they are connected through porous walls), but severely weaken the membranes. Moreover, it is possible to combine zwitterionic materials with common membrane polymers which is an important recent highlight. They do not dominate but progress has been recently made, which opens avenues to more applications in the biomedical field, given the well-established hemocompatibility of some of these materials.

### 2.2. Metal-Based Additives

Apart from organic-based compounds or nanomaterials, inorganic compounds such as metal-based nanomaterials have been investigated to enhance the fouling resistance of membranes against a variety of organic and biofoulants. Common metallic nanomaterials that are utilized for blending modification are shown in Table 2, including metallic or zerovalent metals, biphasic metals such as metal oxides and metal dichalcogenides, and bimetallic oxides.

#### 2.2.1. Metallic Additives

Recently, metallic nanoparticles have been studied as additives in membranes due to their antimicrobial properties. Aside from being non-toxic and hypoallergenic, silver (Ag) nanoparticles have been widely used as an antimicrobial agent in medicine, and also in wastewater treatment. Ag can interact with the sulfur and phosphorous groups, most commonly in thiol groups (S-H) in proteins, which contributes to the disruption of the bacterial proteins and hinders both respiration and electron transfer [232]. Studies have shown that the use of Ag nanoparticles into the membrane matrix significantly enhanced the permeation flux, antimicrobial, and antifouling properties of membranes as a result of increased hydrophilicity [172,173,174,175,176]. However, this is prone to solubility issues in the matrix and is also susceptible to particle aggregation which altogether affects their stability in the matrix. In addition, the long-term use of membranes impregnated with Ag nanoparticles has been greatly challenged by the leaching of Ag, which results in a decline in the antimicrobial and antifouling properties of the membranes. Ultimately, if the leached Ag gets into our drinking water, this could lead to metal poisoning which could lead to fatal consequences [232]. Esfahani recently reported an interesting study on citrate-stabilized gold (Au) nanoparticles for PSf membranes. They reported that the presence of the nanoparticle degraded the cake formed by humic acid which significantly enhanced the antifouling properties of the membrane (*FRR* = 95%). The disaggregation of the HA cake on the surface could be attributed to the ligand exchange interaction between the HA molecules and citrate-stabilized Au nanoparticles. Instead of forming hydrophobic–hydrophobic bonds with the membrane, HA formed hydrogen bonds with carboxyl groups of the nanoparticles. These newly formed bonds altered the existing hydrogen bonds between HA molecules, and therefore changed the hydrophobic forces that hold the HA molecules together [177,233,234,235].

#### 2.2.2. Biphasic Metals

Zirconium oxide (ZrO_2_) is of considerable interest for its outstanding thermal stability, chemical stability, corrosion resistance, and biocompatibility. As such, it can contribute to the formation of more stable and biocompatible membranes applied in harsh environments. The Zr-O bond can be readily hydrolyzed, resulting in the formation of hydroxyl groups, and ultimately increasing the membrane’s hydrophilicity [179,180,181]. Pang et al. fabricated an antifouling MMMs UF membrane by blending ZrO_2_ with PES [179]. The addition of ZrO_2_ increased the pore size and porosity, resulting in the enhancement of the PWF. Moreover, the *FRR* of a modified membrane containing 1.0 wt% ZrO_2_ was 1.8 times that of the bare PES membrane. However, larger surface pore size for the modified membranes resulted in slightly lower rejection performances. Similarly, Huang et al. prepared a UF membrane by blending ZrO_2_ with natural bamboo cellulose (BC) [181]. Compared to the unmodified cellulose membrane, the ZrO_2_/BC membrane was reported to be more stable and showed improved antifouling capabilities. To avoid the aggregation of nanoparticles and also to further increase the surface hydrophilicity, Shen et al. grafted poly(*N*-acryloylmorpholine) (PACMO) on the nanoparticles [180]. The resulting ZrO_2_-*g*-PACMO endowed PVDF membranes with excellent *FRR* (97%) after the separation of oil/water mixtures. The hydrophilic effect of grafted PACMO chains and the well-distributed ZrO_2_-*g*-PACMO in the membrane pore channel allowed for the efficient removal of oil droplets from the membrane pores.

Zinc oxide (ZnO) is one of the low-cost and multifunctional inorganic nanoparticles that is gaining popularity due to its unique features including its catalytic activity and powerful bactericidal effect. Furthermore, because of their high hydrophilicity, ZnO nanoparticles are one of the best materials for improving the hydrophilicity of composite membranes. ZnO nanoparticles can effectively adsorb water molecules and can be readily incorporated into a matrix polymer [82,236,237]. Rajabi et al. blended ZnO of different structures (ZnO nanoparticle, ZnO-NP, and ZnO nanorod, ZnO-NR) with PES to investigate the effects of the nanofiller on the membrane’s properties [82]. The improved hydrophilicity of the modified membranes was confirmed by WCA measurements. ZnO-NP and ZnO-NR showed a WCA of 60° and 54°, respectively, while the virgin PES membrane exhibited a WCA of 77.9°, resulting in improved PWF. The antifouling property, determined by filtering milk powder solution, revealed that ZnO-NR slightly outperformed ZnO-NP. It could be attributed to a better migration of ZnO-NR towards the top surface during membrane formation, hence resulting in more adsorbed water molecules. Rabiee et al. studied the permeability and antifouling properties of PVC UF matrix membranes modified with ZnO nanoparticles [236]. Again, the effect of the nanoparticles on fouling resistance was clearly highlighted, with a sharp increase in *FRR* (from 69.3% to 91.8%) after fouling test (BSA filtration). As for other types of nanoparticles, aggregation may occur as reported by these authors from a loading of 4 wt%, leading to pore blockage. However, a small quantity of ZnO already ensures significant improvement in the overall antifouling properties of membranes, as seen from the results reported by Purushothaman et al., who modified poly(ether ether sulfone) (PEES) membranes and observed a significant improvement of the fouling resistance to HA using as little as 1 wt% nanoparticles [237]. Hence, the *FRR* jumped from about 45% for the pristine membrane to over 92% for the modified membrane.

The use of titanium oxide (TiO_2_) has gained momentum due to its ease of synthesis, stability under extreme conditions, and commercial availability. It is considered to be an outstanding material for the development of composite membranes applied in water treatment as it also shows excellent oleophobicity, which reduces membrane fouling by oily compounds. Furthermore, TiO_2_ nanoparticles are chemically stable, have a high wettability by water, are antimicrobial and can act as catalysts for the degradation of micropollutants [15,38], although we will only focus here on their antifouling properties under normal conditions (i.e., no irradiation promoting catalytic degradation of foulants). Zhang et al. used a nano-TiO_2_/PEG composite to modify PVDF membranes [38]. PEG was primarily utilized in the casting solution to increase the stability and dispersion of the TiO_2_ nanoparticles. Compared to bare PVDF membrane, the pore size and porosity of TiO_2_-modified membranes were decreased. Furthermore, the PWF dropped gradually as the TiO_2_ concentration increased above 0.15 wt%, which was attributed to the excessive aggregation of nanoparticles, which leads to pore blockage during phase separation as well as to an increase in surface WCA and overall poor membrane performances. However, provided that the homogeneous dispersion of the nanoparticles is achieved for a concentration below 0.15 wt%, the surface hydrophilicity of the membranes could be improved (WCA decreased). Arif et al. also fabricated PVDF/TiO_2_ composite membranes [190]. By controlling the TiO_2_/PVDF weight ratio to 0.01/1, they obtained a membrane exhibiting a high *FRR* (approximately 88.2%) after fouling tests, demonstrating the beneficial impact of the nanoparticles of fouling resistance. Again, however, higher contents (0.02 g TiO_2_/g of PVDF) led to the agglomeration of TiO_2_ particles, causing the surface roughness to increase due to the creation of bumps, resulting in poorer antifouling performances. Moghadam et al. also worked with a similar system (PVDF/TiO_2_) and prepared a UF membrane for the removal and degradation of pollutants [238]. While the catalytic effect of TiO_2_ under UV irradiation is beyond our scope, the authors showed a significant increase in PWF and protein flux of the membranes without UV irradiation, hence supporting the improvement of membrane hydrophilicity and reduced membrane fouling resulting from the inclusion of nanoparticles in the matrix.

Silicon oxide (SiO_2_) can be utilized as inorganic modifier of membranes for its stability, strong hydrophilicity, mild reactivity, and high chemical resistance. However, direct mixing with polymers is challenging as it is difficult to obtain well-dispersed nanoparticles in the membrane, limiting their range of application. Thus, their surface modification prior to dispersion is recommended. Tripathi et al. prepared membranes by mixing silica nanoparticles modified with dopamine (DOPA) and polyacrylonitrile (PAN) [239]. The change in performance, hydrophilicity, and antifouling features of composite membranes containing increased concentrations of SiO_2_-DOPA were compared to those of pure PAN membranes. The modified membranes demonstrated increased PWF and rejection capabilities in comparison to the unmodified PAN membrane. SiO_2_ nanoparticles that had been modified with DOPA had greater dispersion in the organic solvent (DMF) during membrane fabrication. The particles were evenly distributed in the solution, and there was no evidence of agglomeration even after extended storage of the solution. As a result, the compatibility of particles with the PAN matrix was improved. The authors also stressed the improvement of *FRR*, and reported both high protein rejection and dye rejection, suggesting their applicability for wastewater treatment.

Copper oxide (CuO) nanoparticles have also been explored recently. It is non-toxic and highly abundant in nature [217]. Hosseini and colleagues [188] reported a decrease in the WCA of PES membranes ascribed to the inherent hydrophilicity of CuO, and subsequent migration on the surface of PES upon phase-inversion. However, a significant increase in WCA occurred as the concentration CuO was increased. This could be attributed to the agglomeration of nanoparticles in the dope solution, and these agglomerated nanoparticles migrated to the surface which resulted in increased surface roughness. The influence of leaching of CuO on the membrane performance was investigated by Kaju et al. [218] in which they found that after exposing the CuO-modified PES membrane to different cleaning solutions (0.1 M NaCl, 0.1 M HCl, 0.1 M NaOH, 0.5% NaClO, and pure water) resulted in increased porosity, enhanced permeability, and decreased salt rejection. Although comparable results were also obtained on pristine PES, no significant increase in porosity on the virgin membrane was observed. Hence, the evident enhancement in the porosity of the modified membranes after long hours of cleaning could be attributed to the leaching of CuO.

Iron oxides in the forms of Fe_2_O_3_ (Ferric oxide) and Fe_3_O_4_ (Ferrous oxide) are promising metal oxides for improving the membrane performance against fouling with improved permeability [14,51,205,219,220,221,222,223,224,225,226,227]. In addition, due to the magnetic nature of iron oxide, it is useful to the study of the photocatalytic performance of polymeric membranes [240]. However, iron oxides have poor solubility in organic solvents [51] and are also prone to agglomeration, as shown in the works of Demirel et al. [223,224]. To resolve this, modifications involving the immobilization of reactive ligands on the surface of nanoparticles, surface coating with an adsorptive layer, or a combination of both, have been recently studied to improve the affinity of iron oxides in the polymer matrix [51], and consequently enhance the membrane performance. For instance, Zinadini et al. [227] blended O-carboxymethyl chitosan coated Fe_3_O_4_ into the PES matrix, resulting in an increased hydrophilicity, dye rejection, and fouling resistance. Kamari and Shahbazi [205] recently reported the incorporation of NH_2_ functionalized SiO_2_ coated Fe_3_O_4_ in PES membranes that led to better membrane performance in terms of permeability, salt rejection, heavy metal ion removal, methyl red dye retention, and resistance towards BSA fouling.

Aluminum oxide (Al_2_O_3_) has also been considered as an antifouling additive for membranes due to its non-toxicity, and affordability [228]. Mehrnia et al. studied the concentration threshold of Al_2_O_3_ in PSf membranes by examining the rheological properties of the dope solution with different Al_2_O_3_ concentration. According to their study, when the concentration threshold is exceeded, a drastic increase in the viscosity of the dope solution is observed which could affect the thermodynamic stability, and kinetics of the phase-inversion process. There is a noticeable drop in the porosity of PSf beyond 0.39 wt% of Al_2_O_3_; however, high permeability is still maintained due to increased surface wettability owing to the hydrophilicity of the nanoparticle [55]. However, it must be noted that despite the intrinsic hydrophilicity of the nanoparticle, further increase in the concentration would not equate to increased surface wettability of the membrane at some point. In fact, the opposite could be observed, as reported in earlier study [229], due to the increased agglomeration of the nanoparticles resulting in increased surface roughness.

#### 2.2.3. Bimetallic Oxides

Bimetallic oxide nanoparticles combine within one material the advantages of several monometallic nanoparticles but exhibit smaller sizes and both higher surface areas and stability. Considering their properties, bimetallic should lead to even better hydration and fouling resistance than monometallic nanoparticles. From these considerations, Arumugham et al. modified PES membranes prepared by LIPS using nanoparticles of iron(III) oxide and manganese (III) oxide [231]. After adjusting the concentration in nanoparticle, they managed to minimize the surface roughness, surface hydrophilicity, and maximize the porosity. Consequently, the optimized membranes exhibited a permeability which was almost double that of the pristine matrix, and fouling was well-mitigated after five cycles of dynamic tests involving BSA (*FRR* of 65.8%).

### 2.3. Ceramic-Based Additives

Ceramic materials are more thermally stable and have greater mechanical strength than polymeric materials. In recent years, the blending of hydrophilic ceramic materials (listed in Table 3) into polymeric matrix membranes has emerged as an efficient method to mitigate fouling.

These nanofillers can change the surface characteristics of membranes and increase their antifouling performance [232], provided that homogeneous dispersion is achieved, as aggregation can lead to structural defects then causing a decline of all performances. Fouling resistance, excellent permeability, good flux recovery, chemical stability, and an extended lifespan are just a few of the benefits of blending ceramic elements into polymeric membranes. Thus, ceramic materials show promising results in water treatment [36,282]. Considerable attention has been given to ceramic nanomaterials such as nanoclays due to their intrinsic hydrophilicity, high surface area, good mechanical strength, low toxicity, and low cost [60,62,255,283,284]. In addition to these, nanoclays could also improve the thermal stability and mechanical strength of the membranes [284]. The nanoclays listed in Table 3 have been shown to endow polymeric membranes with improved surface wettability which evidently enhances the antifouling properties. However, they also suffer from immiscibility in the polymer matrix and agglomeration which consequently decreases the membrane performance and also negatively impacts the mechanical properties of the membrane. In general, inorganic nanomaterials have high surface energy. This, along with high interparticle interactions, is the primary contributor for the agglomeration of nanoparticles. With high concentration or loading, the distance between filler nanoparticles becomes very small and they tend to agglomerate because of the van der Waals forces which prevents the nanoparticles from dispersing uniformly [285]. In order to improve this, these nanomaterials are functionalized with hydrophilic matierials such as taurine (–SO_3_H) and PDA [253], activated carbon [252], folic acid [247], (3-aminopropyl) triethoxysilane (APTES), silane coupling agent (–NH_2_), ethoxy (–OC_2_H_5_) [250], curcumin (–OH, phenols, and carbonyl groups) [260], *N*-halamine (–NH_2_, –C=O) [264], and dextran (–OH) [276] to improve their miscibility and dispersibility in the blend (dope casting solution).

### 2.4. Carbon Allotropes and Porous Nanomaterials

Over the past decades (not limited to membrane technology), carbon allotropes and porous nanomaterials have been tremendously employed in various studies. Lately, these materials have been introduced as upcoming additives for membranes due to their interesting properties such as complex functional groups, high surface area, and so on. Listed below (Table 4) are the different carbon allotropes and porous nanomaterials that have enhanced the antifouling properties of common polymeric membranes through blending approach.

#### 2.4.1. Carbon Allotropes

Carbon allotropes including graphene oxide (GO) and reduced graphene oxide (rGO), as well as other carbon materials such as multiwalled carbon nanotubes (MWCNTs) or carboxylated nanodiamonds (CNDs), have been used in the past decade to develop membranes by blending modification for water treatment [288,302,304,305,306,307,308,344,345,346,347]. 

CNDs, prepared via the thermal treatment of nanodiamond particles in order to incorporate oxygen-containing groups and remove sp^2^ non-diamond carbons, have been reported in the study of Li et al. [288]. The authors showed that adding 1 wt% of CNDs drastically improved the antifouling properties of PVDF membranes. Moreover, they emphasized the role of thermal treatment and showed that membranes modified with raw nanodiamonds still suffered from important fouling. They also pointed to the need for adjusting the concentration in CNDs in the casting solution.

Graphene (GN) is a two-dimensional (2D) material composed of carbon atoms with sp^2^ hybridization orbitals arranged in a hexagonal lattice in a planar configuration. The wetting of GN is thickness dependent, and monolayer GN is hydrophilic, making it a potential candidate for the mitigating membrane biofouling in aqueous media. It has been utilized to construct nanocomposites for several membrane applications [307,344]. Recently, graphene quantum dots (GQDs) modified with citric monohydrate were used in combination with PVC and PEG [308]. The functionalization of GQDs with hydrophilic functional groups improved the antifouling properties provided that careful adjustment of the nanoparticles content. Indeed, the roughness value increased at high concentration (2.0 wt%), causing fouling to increase. The inclusion of oxygen-containing functional groups to graphene such as hydroxyl, carboxyl, carbonyl, and epoxy groups results in the formation of GO, more hydrophilic than GN. Several teams reported that the blending of carbon-based materials into the polymer matrix such as GO [304,305,306] or rGO [306] increases the membrane’s hydrophilicity, resulting in improved permeability during water treatment. Xia et al. incorporated GO into a PVDF membrane prepared via NIPS [305]. They pointed out that GO contributed to the removal of the dissolved organic carbon (DOC) through its oxygen-containing functional groups, allowing the membranes to maintain high flux throughout the permeation process. A low content (0.1–0.3 wt% as in the study of Mohsenpour et al. [304] or 0.5 wt% GO as in the work of Xia et al. [305]) is enough to achieve the desired properties. High amounts of GO have a detrimental impact on the permeability of the membrane, possibly due to a decrease in porosity and pore size resulting from an increased viscosity of the casting solution used to produce the membrane. To boost the performances of membranes, the aggregation of GO nanosheets at high GO concentrations must be avoided. Moreover, not only the GO loading should be fine-tuned, but also the reduction degree, as examined by Meng et al. who reported that a higher dissolution temperature could lead to a lower degree of reduction which could then prevent aggregation [306]. Note also that the presence of hydroxyl groups of GO impacts the kinetic of mass transfers during membrane formation, hence affecting the membrane structure. More or larger macrovoids are likely to be observed if membranes are formed by wet-immersion, as pointed out by Bala et al. [345] or Mohsenpour et al. [304].

MWCNTs are materials composed of multiple graphene sheets organized in a concentric fashion. In water treatment application, they can be used for their ability to adsorb large quantities of micropollutants, property imparted by their large surface area. Nevertheless, they are generally hydrophobic. So, their functionalization with hydrophilic pendant groups before their inclusion in membrane systems is recommended in view of maintaining high membrane permeation. In addition, the grafting of some particular functional groups onto MWCNTs can enhance the interactions, leading to the trapping of pollutants. Mahdavi et al. [303] and then Haghighat et al. [302] synthesized polypyrrole-modified MWCNTs (PPy-MWCNTs), by an oxidation polymerization process, before their inclusion in membrane systems. The existence of amine groups on the surface of the membrane permitted the improvement of the hydrophilicity of the modified membranes, which then led to fouling mitigation. However, as for other carbon-based materials, particle agglomeration may occur at large contents, and the addition of 0.25 wt% of PPy-MWCNTs in the study of Haghighat et al. [302] led to the best outcome in terms of water permeation and dye removal.

So, oxygenated derivatives of graphene and carbon materials have been used in the past ten years in combination with polymeric materials and have proven to be an efficient way to control surface wettability, enhance rejection performances and mitigate fouling of numerous polymeric membranes prepared by phase-inversion. Nevertheless, the preparation of homogeneous casting solution remains a challenge that need to be addressed, and it is difficult to use optical techniques to assess the particles size or the extent of particles aggregation. Yet, this phenomenon must be prevented as it also weakens the membranes. Furthermore, these materials significantly increase the casting solution viscosity, which then has consequences on the membrane structure and arising performances.

#### 2.4.2. Porous Nanomaterials

Porous nanomaterials such metal-organic framework (MOF), Zeolites/ZIF, and cyclodextrin are some of the emerging nanomaterial additives to help resist the adsorption of non-specific foulants. These nanomaterials have highly porous structure and high surface area which could help improve the porosity and flux performance of the membranes. The organic linkers of these nanomaterials could help induce their uniform dispersion in the casting solution. Aside from providing good antifouling abilities to the host matrix as reported in the studies in Table 4, MOF and zeolites/ZIF have good photocatalytic properties towards emerging pollutants such pharmaceutical foulants [348].

#### 2.4.3. Concluding Remarks on the Use of Inorganic Nanoparticles (Metal-Based, Ceramic-Based, Carbon Allotropes and Porous Nanomaterials)

In general, the nanofiller’s hydrophilicity helps to improve the wettability of the modified membranes, ultimately resulting in improved antifouling property of the composite membranes. Compared to polymer additives, smaller concentrations are enough to significantly improve the antifouling properties of membranes. Furthermore, higher rejections as the additive content increases are commonly reported due to the increase in the casting solution viscosity leading to denser membranes. Moreover, some nanoparticles can reduce fouling because of their intrinsic hydrophilicity, but also, because they act as catalyst (although beyond the scope of this review). However, high concentrations cause the aggregation of nanoparticles, which blocks surface pores, reduces the membrane performances, and even sometimes their antifouling characteristics.

Similar to polymeric additives, leaching and aggregation are some of the major drawbacks in using inorganic additives such as metals. However, it appears to be exacerbated in inorganic additives due to its incompatibility with the polymer matrix. The use of physical blending of these nanomaterials in the polymer casting solution and phase-inversion gave rise to the poor distribution of the nanomaterials in the matrix as a result of the formation of aggregates in the dope casting solution. To overcome these problems, researchers have tried to increase the surface area of the nanomaterials by controlling their particle size [33]. Another method is functionalizing the nanomaterials in order to increase the interaction with the host material [204]. These observations reported by several groups, regardless of the nature of the nanoparticles, suggests the need for optimizing the composition of the casting solution before phase separation. Materials such as PEG, DOPA, etc. can be added to the blend, which main function is to limit aggregation and improve dispersion. In order to achieve the latter, the precursors of the nanomaterials must be soluble in the dope casting solution and can react with the non-solvent in the coagulation bath [34,349].

### 2.5. Hybrid Nanomaterials

Recently, the development of new antifouling materials has involved complex synthesis of additives. Some studies combine two sets of additives and blend them together into the polymeric matrix [81,289,350,351,352,353,354] to improve both antifouling properties and photocatalytic activities of the prepared membranes. However, this should be carried out with great consideration with regards to the ensuing thermodynamics instability of the system, and how this could also influence the viscosity and kinetics of the phase separation. Moreover, we should be reminded that this could lead to high degree of aggregation in polymeric matrix, instead of improving the antifouling properties, this could eventually lead to the poor performance of the membranes in terms of resisting foulants, and separation performance. Additionally, poorly dispersed additives could aggravate leaching.

## 3. Blending of Additives into the Polymer Matrix and Its Effect on Membrane Morphology

As mentioned earlier, blending modification refers to the blending of the main polymer with an antifouling material in a common solvent, prior to applying a phase-separation technique. There are three major phase-inversion processes leading to the formation of porous membranes (LIPS (wet-immersion), VIPS, and TIPS) with which blending modified membranes can be prepared. Although the purpose of the modification is to impart the membranes with antifouling properties, it also impacts membrane structure. Below is a discussion on major effects observed based upon recent literature.

### 3.1. Influence of the Antifouling Additive on the Morphology of Liquid-Induced Phase Separation Membranes

The fundamentals of membrane formation by phase-inversion have been thoroughly described [355,356] and are beyond the scope of this text. Instead, we would like to discuss the effects of the additive on thermodynamic and kinetic based on experimental observations published in the last 10 years.

**Figure 4:**
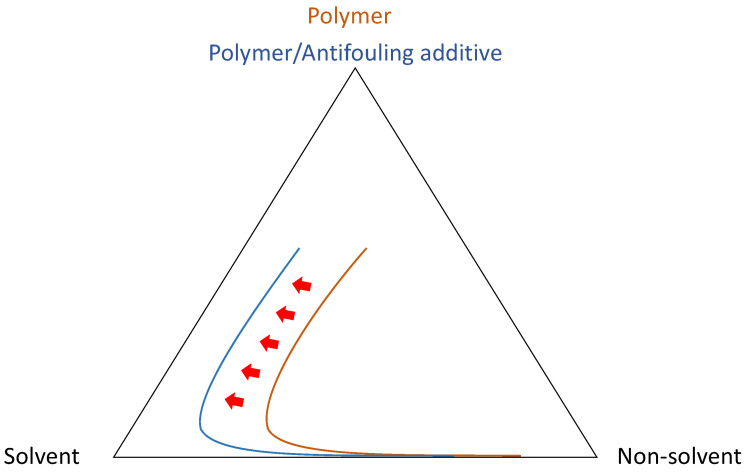
Schematic effect of the antifouling additive on the position of the demixing gap. Red arrows highlight the shift of the binodal line towards the solvent/polymer axis.

Thermodynamics is described by the Flory-Huggins theory [355]. In this theory, the binodal line separates a single-phase region from a two-phase region. It can be determined experimentally by cloud points measurements, which consists of adding dropwise non-solvent to a casting solution under constant stirring and at constant temperature and measuring the total volume of non-solvent needed to induce permanent cloudy state. For this composition, phase separation has been reached. It can then be positioned in a ternary phase diagram represented by a triangle, with each vertex representing a pure component of the system: polymer, solvent of non-solvent. Adding an antifouling material to the casting solution complexifies the analysis since the composition of the quaternary system (polymer/antifouling material/solvent/non-solvent) should be plotted in a 3D diagram (triangular prism). However, it is not carried out as the third dimension is already taken by the temperature (a ternary phase diagram is only valid at one given temperature). Instead, a pseudo-ternary phase diagram is determined where the polymer and antifouling material may occupy one same vertex of the triangle. So, in this representation, polymer and antifouling material are seen as one unique material. In doing so, the polymer/antifouling material weight ratio in all of the solutions used to determine the set of cloud points should remain constant. As then observed using this experimental approach and tentatively schemed in Figure 4, the addition of an antifouling material to a casting polymer/solvent solution results in the shift of the binodal line towards the polymer-solvent axis of a ternary phase diagram [86,116,117]. In other words, the demixing gap is reduced such that fewer amount of non-solvent is needed to induce phase-separation. As the system is less thermodynamically stable by the addition of an antifouling material, film formation is facilitated. Logically, if the distance to cross the demixing gap is changed, the time needed to reach the demixing region will be affected as well. Shorter times can be expected although changes in the system viscosity also need to be accounted for.

**Figure 5:**
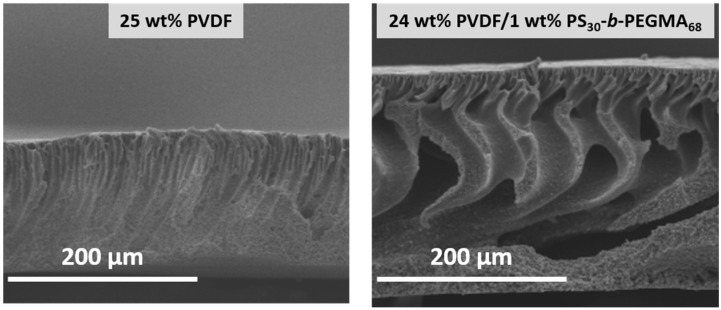
Influence of the addition of 1 wt% PS_30_-*b*-PEGMA_68_ copolymer to a casting solution containing PVDF (NMP as solvent) on the cross-sectional morphology of LIPS membranes (T_dissolution_: 40 °C; Solvent content fixed: 75 wt%; non-solvent: DI water; casting thickness: 300 µm). Left image partially reproduced with permission from [357]. Copyright 2014 Elsevier.

As for kinetic aspects, instantaneous phase-inversion and delayed demixing are often associated with high porosity membranes decorated with macrovoids and with denser, macrovoid-free membranes, respectively. Phase-inversion in LIPS (wet-immersion) often happens rapidly as DI water is a common non-solvent with high affinity with most solvents used in the industrial scale production of membranes, leading to fast exchanges. The addition of hydrophilic of amphiphilic additives increase the affinity of the dope with water, likely promoting exchange rates [357]. Nevertheless, it also affects the viscosity of the system. If an increase in viscosity is measured (seen if few percent of solvent is replaced by the additive, compared to the formulation of the pristine dope), then it may slow-down the exchange rates. Two phenomena need to be accounted for when rationalizing the kinetic of phase-inversion of blending modified membranes: the affinity of the additive with water which is normally high because hydrophilicity is a criterion for antifouling, and the change in viscosity associated with the addition of water. The latter one has often been mentioned in recent literature to explain the disappearance of typical macrovoids observed in LIPS membranes. For example, Khalil et al. developed PLA-based membranes using PVP as an additive [358]. They reported a gradual disappearance of macrovoids with PVP because the additive increased the dope viscosity. Similarly, Nainar et al. [359] mixed PVDF with CA and added PVP to the dope solution. The pristine PVDF membrane possessed macrovoids, which tend to vanish with the addition of hydrophilic CA, a consequence of the higher solution viscosity. PVP then led to larger macrovoids (which could be a consequence of the large affinity with water) although a rather dense sublayer sitting on top of the large macrovoids decorating the cross-sections was observed due to the high viscosity of the casting solution. As another example, macrovoids became much larger with the addition of PS-*b*-PEGMA to a PVDF/N-methylpyrrolidone dope in one of our previous studies [357] due to both the decrease in dope viscosity and the affinity of PEGMA units with water (Data comparing a virgin PVDF membrane and a membrane containing 1 wt% copolymer obtained with a similar system are displayed in Figure 5). Yuan et al. also reported accelerated phase-inversion with the addition of polyvinyl alcohol (PVA) to a polyethersulfone (PES) membrane, resulting in larger pore size and porosity [106]. The effect of viscosity on membrane structure following the addition of nanoparticles is also reported. Nano-SiO_2_ particles have been shown to increase the thickness of the top layer of polyvinyl chloride (PVC) membranes, attributed to a more viscous nano-SiO_2_/PVC/solvent casting solution, compared to a PVC/solvent casting solution [209]. Xia and Ni reported the interplay between viscosity and affinity with water, in a PVDF/graphene oxide (GO) system [305]. The viscosity of the dope increased with the addition of GO, but only 1 wt% led to a sharp increase. At 0.5 wt% and below, the change in viscosity was small. They observed the formation of larger macrovoids in the membrane containing 0.5 wt% GO (compared to the pristine membrane), which they attributed to the affinity of the additive with water, which led to thermodynamic instability, and promoted faster mass transfers. On the other hand, they also pointed out that the porosity of a membrane formed after adding 1 wt% GO decreased (compared to that of a membrane containing 0.5 wt% GO), which they attributed to a larger viscosity.

### 3.2. Influence of the Antifouling Additive on the Morphology of Vapor-Induced Phase Separation Membranes

The VIPS method has been used much less than the LIPS process to fabricate antifouling membranes. In the past 10 years, few teams have worked on the design of such membranes [90,116,117,119,122,360,361,362]. VIPS leads to MF membranes, which makes it a complementary method to LIPS which generates UF membranes. However, the equipment needed is more complex to implement since a control atmosphere of non-solvent needs to be created. Furthermore, it competes with TIPS in terms of pore size range. Yet, the slowness of mass-transfers makes it an interesting process to control membrane structuring, and to optimize surface segregation of the antifouling material. Moreover, there is no initial fast solvent outflow that could readily drag the hydrophilic/amphiphilic additives out of the film before phase-inversion is complete as in LIPS. Thus, the majority of additive molecules is expected to remain trapped in the film.

**Figure 6:**
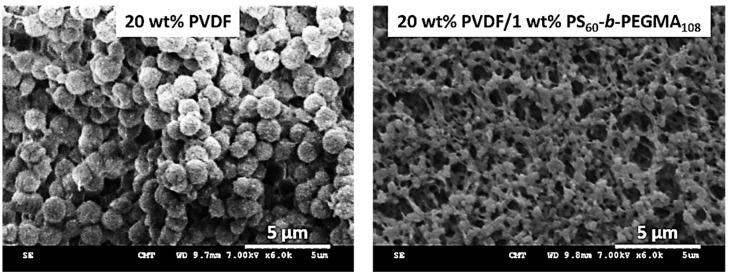
Influence of the addition of 1 wt% PS_60_-*b*-PEGMA_108_ copolymer to a casting solution containing 20 wt% PVDF (NMP as solvent) on the surface morphology of VIPS membranes (T_dissolution_: 32 °C; RH: 70%; T_chamber_: 25 °C; t_exposure_: 20 min; casting thickness: 300 µm).

As in LIPS, the additive has a considerable impact on membrane formation. From a thermodynamic perspective, the impact of the additive on phase-inversion is the same in VIPS and in LIPS, in that it shifts the binodal line closer to the polymer/solvent axis. Likewise, one can expect a similar trend regarding the effect of the additive on formation kinetic, although it is tricky to measure in a VIPS chamber. Again, the measured effects of the additive on the kinetic of phase-inversion cannot just be attributed to the hydrophilic groups in the additive, as the change in viscosity must be considered as well. Carretier et al. reported faster transfer in a system containing 20 wt% PVDF and 5 wt% PEGMA-*b*-PS-*b*-PEGMA than in a system containing 25 wt% PVDF [86] which was not just due to hydrophilic PEGMA, but also to the lower viscosity of the solution containing the antifouling additive. However, Dizon and Venault also reported faster phase-inversion in a solution containing an amphiphilic additive than in a pristine solution. Yet, the former solution was slightly more viscous [117]. In many of our previous reports on the design of antifouling PVDF membranes by VIPS, we noted that the additive helped in preventing nodule formation or at least at decreasing their size [116,117,357,363] (Figure 6). Nodules arise from a crystallization-gelling process [364], and their presence in fouling-resistant PEGylated PVDF membranes formed by VIPS has also been reported by another group [362]. They tend to weaken the membrane structure, but their size can be controlled by the temperature of dissolution of the casting solution. Nevertheless, for a same level of temperature, the amphiphilic additive prevents or at least reduces the growth of nodules. There are two underlying reasons. Firstly, the acceleration of non-solvent transport resulting from the addition of hydrophilic units favors L/L phase separation over crystallization. Secondly, in the case where the viscosity increases after the addition of antifouling copolymer, viscous forces oppose the growth of crystal nuclei. As a consequence, bi-continuous structures are readily formed. These structures are attractive in MF membranes for their combination of high permeability and mechanical stability.

### 3.3. Influence of the Antifouling Additive on the Morphology of Thermal Induced Phase Separation Membranes

The TIPS method has undeniable advantages over the two NIPS processes in that it leads to stronger membranes with a uniform pore structure. Nevertheless, the need for high temperature of the dope is detrimental to its usage for the widespread formation of antifouling membranes by blending modification. In particular, antifouling polymers lack thermal stability. There are plenty of studies starring the TIPS method followed by a post-treatment imparting the membrane with antifouling properties, but very few exist, to our knowledge, in which only TIPS is used to induce phase separation of a polymer/antifouling material/diluent blend. In the period of time on which this survey focusses, we shall mention the work performed in Matsuyama’s group who entrapped a PEGylated copolymer in PVDF TIPS membranes in one step (hollow fibers) [365] as well as those of Xu et al. [23] and Li et al. [366], who used multi-wall carbon nanotube and nanoparticles of SiO_2_ and GO, respectively, to fabricate PVDF membranes by TIPS (flat sheet). Regarding the former, the authors did not notice any significant effect of the copolymer on the membrane structure. All membranes, pristine and PEGylated, exhibited a spherulitic porous structure, with similar roughness, characteristic from the TIPS process applied to a PVDF/diluent blend. The authors justified the absence of significant morphological change with the molecular weight of the copolymer. According to them, it corresponded to an effective diameter too small, in comparison with the surface pore diameter, to affect the membrane structure. However, in the case of MMMs, the authors noticed the role of the nanoparticles on the crystallization of the polymer [23,366]. While the MMMs exhibited a morphology characteristic from L/L phase-inversion (cellular pores/sponge-like morphology), the authors explained that the nanoparticles acted as nuclei promoting the crystallization of PVDF and so the formation of small spherulites [23]. Larger amounts of nanoparticles then led to more crystalline grains but also smaller pores. Eventually, increasing further the nanoparticles content resulted in denser membranes as the dope viscosity increased.

## 4. Assessments of Antifouling Properties of Modified Membranes through Blending Approach

The development of antifouling membranes is complicated by the fact that various fouling scales, such as the nano-scale for proteins and the micro-scale for bacteria, must be considered. If the formation of a strong hydration layer is accepted as a pre-requisite to prevent fouling of membranes used in aqueous media, the characteristic size of foulants needs to be considered. As wastewater or biological media often contain a complex mixture of proteins and cells as biofoulants, both should be employed to assess the fouling-resistance of a membrane. Some of the major tests run by the authors of the studies relevant to this review are summarized below, and some essential membrane characteristics and performances listed in Table 5. From this table, we could clearly see an increasing trend in the antifouling properties of the modified membranes as their hydrophilic properties were enhanced, which essentially constituted the increased rejections of various foulants, and overall better performance.

### 4.1. Hydrophilicity Tests

As it is for dense interfaces, hydrophilicity is an important factor in determining the ability of a porous membrane to resist fouling [367]. The water contact angle (WCA), hydration capacity (HC), and strength of the hydration layer of the membranes may be measured to characterize the surface hydrophilicity, its ability to trap water, and the stability of the hydration layer, respectively.

WCA is commonly measured with a contact angle analyzer instrument employing the static sessile drop technique. It is likely the most employed test as it is readily carried out. Moreover, it can be dynamically monitored, that is, the evolution of WCA with time can be tracked, which is useful when wetting is not instantaneous. It has been recently reported that with VIPS PVDF membranes, the WCA could be decreased by 15–20° within 80 s [123]. This is associated with the intrinsic hydrophobicity of the PVDF material enhanced by the particular morphology of PVDF membranes prepared by VIPS, which are highly porous and rough, two properties that contribute to prevent or delay wetting. In general, lowering the surface roughness facilitates wetting, and so, permits the reduction in the adhesion forces between the membrane and foulants [190,368,369]. The WCA of PVDF membranes prepared by VIPS [123] is much larger than that of similar membranes obtained by LIPS [357], clear evidence of differences in surface structuring. Hence, some “induction” time may be required for the water molecules to interact with the antifouling moieties on the surface of such rough membranes. Though the WCA provides intelligence on the surface hydrophilicity, one may question its actual relevance when membranes are to be contacted with aqueous medium for some extended period of time, typically in wastewater treatments. As just discussed, the WCA of VIPS membranes, although modified, remains high. Yet, they are able to trap a significant amount of water after prolonged contact. This suggests that water can penetrate and wet the pores of the membrane, provided that it has been modified. Although surface segregation is known to occur during the preparation of antifouling membranes by phase-inversion methods [370], these processes maintain bulk modifications which means that a significant proportion of the antifouling additive is trapped inside the matrix. These trapped antifouling moieties contribute to water trapping in the bulk and are also likely to reduce fouling inside the membrane. Hydration capacity tests or swelling tests, which are gravimetric tests, are readily performed and can provide relevant related information. These tests do not require any equipment other than a precision balance with which the weight of the samples before and after immersion in water are measured. Then, one can report the amount of water trapped per unit volume of the membrane (hydration capacity) or normalized using the dry weight the difference between the weight of the hydrated membrane and the weight of the dry one (swelling ratio also sometimes referred to as equilibrium water content). This will likely provide a useful assessment of the hydrophilicity of a porous membrane. Finally, it is worth mentioning that the strength of the hydration layer can help in discussing the antifouling properties of a membrane. Following the work of Ishihara’s group [167], we have been evaluating the strength of the hydration layer of some blending modified membranes using differential scanning calorimetry measurements [90,123]. These tests, combined with the results of hydration tests, permit the determination of the proportion of non-freezable water. The higher it is, the stronger the hydration layer. Mapping FT-IR can also be used to assess hydration differences between samples which correlates to the strength of the hydration layer. We used this approach after drying samples, to evaluate whether water could still be detected on the surface of membranes, and to compare several antifouling additives [117]. It is more of a qualitative assessment but still permits the highlighting of clear differences regarding the ability of antifouling materials to retain water, and also proved that zwitterionic materials performed better than PEGylated ones.

### 4.2. Static Fouling Tests

Static fouling tests may be conducted, as carried out for model dense interfaces through which no permeation occurs. These tests require the incubation of the membrane samples with a solution containing the foulants (typically proteins or cells). They mostly permit the evaluation of the surface antifouling properties. If proteins are used, the advantage over dynamic tests is that they are simpler to implement and require lower volumes of solutions. This also significantly lowers the costs when human plasma proteins such as fibrinogen (FN), human serum albumin or γ-globulin are used to test the antifouling properties of biomedical membranes [41,361]. Furthermore, because cells such as gram-negative bacteria or human blood cells can be easily deformed under a pressure gradient sometimes resulting in cell lysis, carrying static adsorption tests eliminates the influence of pressure, and permits us to solely focus on the material–biofoulants interactions.

**Table 5:** Some performances of blending modified membranes reported in the past decade.

Class of Additive	Matrix Polymer	Antifouling Additive	Process	Water Contact Angle (^o^)		Pore Size (nm)		Porosity (%)		Pure Water Permeance * (L/m^2^·h·bar)		Flux Recovery Ratio (%)		Foulant Adsorption ^a^ or Rejection ^r^ (% or (μg/cm^2^) ^d^)		Ref.
				Virgin	Modified	Virgin	Modified	Virgin	Modified	Virgin	Modified	Virgin	Modified	Virgin	Modified	
Polymer/organic-based	CA	PAA	LIPS	71.5	25.0	12.9	9.6	44.6	75.6	15.3	19.0	88.2	97.6	^r^ HA (95.7)	^r^ HA (99.9)	[108]
	PEI	PVP	VIPS	96.4	56.1	28.5	50.4	4.2	13.6	4.8	36.8	69.6	81.0	^a^ BSA (170.0) ^d^	^a^ BSA (80.0) ^d^	[94]
	PES	CNC	LIPS	66.0	43.0	12.0	9.0	-	-	33.3	60.9	51.0	90.0	^r^ BSA (93.0)	^r^ BSA (97.0)	[100]
	PES	PAN	LIPS	68.4	56.8	-	-	-	-	24.6	55.6	57.9	86.1	^r^ BSA (93.8)	^r^ BSA (87.1)	[371]
	PES	PAN/PEG	LIPS	68.4	-	-	-	-	-	24.6	202.2	57.9	90.7	^r^ BSA (93.8)	^r^ BSA (81.4)	[371]
	PES	PVA	LIPS	79.0	63.0	11.2	34.9	54.2	65.5	-	131.5	52.0	92.6	^r^ BSA (81.0)	^r^ BSA (61.2)	[106]
	PES	PVP	LIPS	-	-	-	-	-	-	^c^ 72.0	1219.5	^c^ 80.8	98.5	-	^r^ HA (95.0)	[43]
	PLA	PVP	LIPS	82.1	34.1	88.6	42.9	79.2	57.1	185.7	19.3	57.0	93.0	^r^ BSA (57.0)	^r^ BSA (92.0)	[358]
	PSf	PEG400	LIPS	87.7	79.8	-	-	18.7	48.4	0.8	420.0	-	-	- -	^r^ BSA (90.0)/ ^r^ Pepsin (73.0)	[42]
	PSf	zP(S-*r*-4VP)	LIPS	120.0	100.0	6.3	10.4	73.2	74.1	5.7	11.7	40.0	63.0	^r^ BSA (80.8)	^r^ BSA (95.6)	[120]
	PSf	PEGMA	VIPS	63.0	27.0	9.3	10.8	80.8	73.6	110.0	512.0	67.6	84.5	^a^ BSA (15.1) ^d^	^a^ BSA (4.0) ^d^	[372]
	PVC	Lignin	LIPS	106.7	41.5	19.7	25.5	76.0	84.9	111.6	347.7	20.4	92.3	^r^ HA (73.0)	^r^ HA (98.9)	[142]
												38.1	81.3	^r^ Oil (68.1)	^r^ Oil (97.4)	
	PVDF	SPANI	LIPS	92.0	29.0	-	-	-	-	97.2	160.0	66.2	99.2	^r^ BSA (90.0)/^a^ BSA (30.0) ^d^	^r^ BSA (95.0)/^a^ BSA (3.0) ^d^	[124]
	PVDF	CNC	LIPS	81.3	74.0	84.5	155.0			9.8	206.9	71.6	82.5	^r^ BSA (83.3)	^r^ BSA (88.2)	[98]
	PVDF	MPC-derivative	VIPS	137.3	113.7	108.1	155.8	72.0	76.3	^c^ 1087	1143	^c^ 17	42	-	-	[122]
	PVDF	MPC-derivative	VIPS	137	114	30	20	72	70	^c^ ≈900	≈900	^c^ 17	38	-	-	[373]
	PVDF	PS-*b*-PEGMA	LIPS	85.0	-	25.5	51.8	64.3	79.8	43.3	58.3	56.0	91.0	^a^ FN (100.0)/^a^ γ-globulin (100.0)/^a^ HAS (100.0)	^a^ FN (19.0)/^a^ γ-globulin (35.0)/^a^ HAS (29.0)	[41]
	PVDF	PS-*b*-PEGMA	VIPS	122	118	260	820	72	79	5000	13,000	≈10	≈82	^a^ FN (100.0)/^a^ BSA (100.0)/^a^ LY (100.0)	^a^ FN (≈30)/^a^ BSA (≈5)/^a^ LY (≈15)	[153]
	PVDF	PS-*b*-PEGMA	VIPS	122	118	260	820	72	79	5000	10,000	≈25	≈76.9	^r^ MA (>99.7)	^r^ MA (>99.7)	[153]
	PVDF	PVA	LIPS	72.2	69.0	25.3	100.4	62.8	57.6	38.1	47.5	83.0	86.0	^r^ BSA (35.0)	^r^ BSA (5.2)	[107]
	PVDF	PVDF-*g*-PSBMA	LIPS	89.0	67.0	-	-	-	-	121.9	239.1	51.5	81.2	^a^ BSA (110.0) ^d^	^a^ BSA (40.0) ^d^	[118]
	PVDF	PS-*r*-PEGMA	VIPS	140.0	47.0	583.0	513.0	75.4	71.9	-	-	16.0	29.0	^r^*E. coli* (92.5)	^r^*E. coli* (99.0)	[374]
	PVDF	PS-*r*-PEGMA	TIPS	135.7	70.0	≈210	≈210	-	-	≈2400	≈2400	≈45	74.0	^a^ BSA (75.0)	^a^ BSA (18.0)	[365]
	PVDF	SM-derivative	VIPS	145.0	67.0	510.0	430.0	57.0	62.0	≈9000	≈12,000	36.0	90.0	^a^ BSA (100)	^a^ BSA (35)	[363]
	PVDF	PS-*r*-PEGMA-*r*-PSBMA	VIPS	129.0	102.0	140.0	70.0	66.5	72.9	1223.0	1146.0	66.0	91.0	^a^ BSA (100)/^a^ FN (100)	^a^ BSA (12)/^a^ FN (15)	[117]
	PVDF	PS-*r*-PEGMA-*r*-PSBAA	VIPS	140.0	112.0	≈299	≈80	67	55	^c^ 2000	1100	53	73	^r^*E. coli* (83.5)/^a^ FN (100)/^a^ blood (100)	^r^*E. coli* (89.6)/^a^ FN (20)/^a^ blood (20)	[123]
	PVDF	PMAA-*r*-PEGMA-*r*-SBMA	VIPS	139.0	90.0	560.0	150.0	70.0	62.0	^c^ 2500	900.0	-	54.0	^a^ FN (100)/^a^ *E. coli* (100)/^a^ blood (100)	^a^ FN (12)/^a^ *E. coli* (5.6)/^a^ blood (5.1)	[116]
	PVDF	zP(S-*r*-4VP)	VIPS	≈143	≈132	250	100	≈75	≈60	^c^ ≈625	≈1900	12.0	69.0	^a^*E. coli* (100)/^a^ blood (100)	^a^*E. coli* (15)/^a^ blood (10)	[119]
Metal-based	BCM	ZrO_2_	LIPS	41.9	33.6	36.5	39.3	77.3	79.8	286.1	321.5	65.0	90.6	^r^ BSA (71.6)	^r^ BSA (91.2)	[181]
	PAN	SiO_2_-DOPA	LIPS	68.0	32.0	-	-	51.7	77.1	426.7	1075.0	49.0	75.0	^r^ BSA (94.0)	^r^ BSA (98.8)	[239]
	PES	Fe_2_O_3_-Mn_2_O_3_	LIPS	73.0	67.0	40.0	45.5	58.0	74.0	208.0	396.0	64.0	77.0	^r^ BSA (97.0)	^r^ BSA (96.0)	[231]
	PES	ZrO_2_	LIPS	73.6	52.3	-	-	-	-	8.2	83.6	54	97.2	^r^ BSA (97.2)	^r^ BSA (92.7)	[179]
	PES	ZnO-NP	LIPS	77.9	60.0	-	-	61.0	69.0	7.8	12.0	39.0	74.1	-	-	[82]
		ZnO-NR	LIPS	77.9	54.0	-	-	61.0	71.0	7.8	12.5	39.0	70.2			
	PVC	ZnO	LIPS	67.5	54.5	9.3	12.1	67.9	79.8	106.5	201.0	69.3	91.8	^r^ BSA (90.2)	^r^ BSA (97.5)	[236]
	PVDF	SiO_2_@GO	TIPS	-	-	41.2	20.1	-	-	268.5	182.6	48.0	95.0	^r^ BSA (63.5)	^r^ BSA (91.7)	[366]
	PVDF	TiO_2_/PEG	LIPS	74.4	69.0	87.0	86.0	52.9	48.6	72.8	73.1	-	-	-	-	[38]
	PVDF	TiO_2_	LIPS	85.4	70.2	-	-	-	-	158.0	350.0	47.5	88.2	^r^ BSA (57.0)	^r^ BSA (95.0)	[190]
	PVDF	ZrO_2_-g-PACMO	LIPS	93.0	66.0	31.6	17.0	77.6	58.4	36.2	82.4	38.0	97.0	^r^ Oil (74.9)	^r^ Oil (99.9)	[180]
Carbon allotropes	PEES	GO	LIPS	96.4	72.3	45.6	72.7	43.2	65.6	30.7	53.9	62.5	83.2	-	-	[345]
	PES **	GO	LIPS	91.0	67.0	3.0	5.1	52.1	58.5	25.0	225.0	-	-	-	-	[304]
		GO	LIPS	85.0	72.0	6.6	8.6	29.1	61.0	239.6	305.2					
	PVC	PP-MWCNTs	LIPS	67.7	61.2	4.2	4.3	77.5	84.0	30.0	37.5	64.8	70.6	^r^ BSA (96.0)	^r^ BSA (98.0)	[302]
	PVC	GQDs	LIPS	65.0	73.0	2.84	3.01	57.2	55.3	12.2	19.1	68.8	>80.0	^r^ BSA (>98.0)	^r^ BSA (>98.0)	[308]
	PVDF	CNDs	LIPS	76.8	65.3	23.8	33.1	47.6	63.7	49	171	35	85	^r^ BSA (>85.0)	^r^ BSA (>95.0)	[288]
	PVDF	GO	LIPS	71.0	70.0	70.0	115.0	47.5	52.5	137.0	203.5	-	-	-	-	[304]
	PVDF	GO	LIPS	74.2	70.2	484.0	1034.0	59.0	80.0	47	94	-	-	^r^ DOC (8.65)	^r^ DOC (11.30)	[305]
	PVDF	GO/TiO_2_	LIPS	79.0	61.0	48.1	65.2	69.6	83.1	158.1	487.8	43.0	71.1	^r^ BSA (80.0)	^r^ BSA (92.5)	[369]
	PVDF	O-MWCNT	TIPS	106.8	98.0	-	-	84.5	83.8	270.7	164.5	-	82.7	^r^ BSA (68.5)	^r^ BSA (90.8)	[23]

^a^ adsorption; ^c^ commercial membrane; ^d^ μg/cm^2^; ^r^ rejection. * Often determined at 1 bar. However, some researchers made use of different transmembrane pressure and the overall pure water permeances were normalized. The table aims at comparing the performances of the pristine and modified membranes under similar operating conditions. So, one is invited to refer to the cited work for more details regarding the experimental protocols. ** Different solvents were used.

#### 4.2.1. Protein Adsorption Tests

BSA is frequently used for static (and dynamic) tests [238,357,365,372] because it is inexpensive. Moreover, it is stable in water-miscible polar solvents, and it is biodegradable [160,375,376,377]. As it possesses absorption peaks in the ultraviolet region, its concentration in the incubation solution can be tracked with a UV-visible spectrometer. Then, a mass balance permits the evaluation of the amount of protein that interacted with the membrane samples. It is somewhat an indirect evaluation of biofouling. A more direct visualization of protein adsorption in the membrane has been reported by the group of Matsuyama who prepared blending modified TIPS membranes [365]. The method consists of utilizing a fluorescent derivative of BSA (fluorescein isothiocyanate conjugate BSA or FITC-BSA). In doing so, the protein can also be directly visualized with a fluorescent microscope or a confocal microscope, and an image analysis software can help in quantifying the change in fluorescence intensity. In our group, we have used other proteins such as lysozyme (LY) [357,361] or fibrinogen and other plasma proteins [41,361]. The method with LY is similar to that involving BSA. LY was used to evaluate the effect of the protein Mw on biofouling. For FN and plasma proteins, an enzyme-linked immunosorbent assay is performed. These plasma proteins are used when membranes are intended to be applied in biomedical devices, in contact with blood, as the early adhesion of FN can then trigger mechanisms of blood cells adhesion and blood coagulation. Therefore, it is relevant to use these proteins as model biofoulants rather than BSA.

#### 4.2.2. Cell Attachment Tests

Bacteria can be utilized to study the interactions between large biofoulants and the membrane material in static conditions. As some can be potentially harmful, the advantage of using static tests over dynamic filtration tests is that volumes needed are much smaller in the former case. Small membrane samples can be positioned in well-plates and then incubated with <1 mL of bacterial solution. Then, the well-plate can be disposed of. Contrariwise, large volumes of bacterial solution would be needed for filtration tests, which would then require careful cleaning and disinfection of the device. Bacteria of different sizes or shapes and belonging to the class of gram-positive or gram-negative have been used in such tests [373] although *Escherichia coli* is the most employed microorganism for testing the antifouling/antibacterial properties of membranes [209,357,378]. Solid culture medium containing agar can then be employed to quantify the resistance to bacteria of the modified membrane, relative to the pristine one [209,361]. The extent of bacterial attachment can be visualized by various microscopy techniques including SEM [209,357], but also by fluorescence microscope or confocal microscope [122,373]. Genetically modified bacteria with a fluorescent protein have been used, which facilitates the sampling before observation since staining is unnecessary [122]. Moreover, different incubation times of the samples with the bacterial medium may provide information on the resistance of the membranes to biofilm formation.

Regarding blending modified membranes with potential applications in blood-contacting devices modified with zwitterionic materials [116,117,122,123], static adsorption tests making use of platelet, red blood cell or white blood cell concentrates, but also whole blood, can be implemented. These tests provide useful information associated with the hemocompatibility of the membrane surface. With whole blood or platelet concentrates, lack of blood compatibility of the membrane can be visualized by SEM by the formation of a fibrillar network arising from platelet activation [117]. As red blood cells concentrate alongside the incubation medium, numerous cells of typical biconcave shape can be observed if the membrane surface is not slippery. It can be interesting to use confocal microscope (again providing fixing/staining of the cells with a glutaraldehyde solution and a solution of 4′,6-diamidino-2-phenylindole) and extract 3D images to locate the position of the cells. In one of our early works, we used this technique that revealed that the cells were not all located on the membrane surface, but instead, managed to penetrate in the deeper layers of the membranes despite the lack of a pressure gradient [361]. Although the mechanism is unclear, the large pores of VIPS membranes, associated with the flexibility of the cell wall of the cells certainly contributed to their penetration and detection beneath the surface.

### 4.3. Dynamic Fouling Tests

The majority of works on the development of antifouling membranes showcases antifouling performances during filtration. This makes sense since membranes are almost only used to separate species during filtration. Thus, blending modified porous membranes, which were the key focus, are no exception. In Table 1, we listed the *FRR* of membranes, obtained after one or several water/biofouling solution/water filtration cycles. As also listed in the references, most studies report BSA protein as the model biofoulant. This can be explained by its availability at low-cost, its safety-in-use compared to bacterial solutions or blood solutions, but also by its suitability to be rejected by ultrafiltration membranes. Undeniably, the most employed process to prepare blending modified membranes is LIPS which commonly leads to membranes falling in the UF range. For all of these reasons, using BSA is appropriate. Nevertheless, there is no standard procedure for studying fouling during filtration. For example, Li et al. performed three complete water/BSA filtration cycles using a 0.5 g/L BSA solution [288] while Yuan et al. performed one cycle using a 3 mg/L BSA solution [106]. In other works, Arumugham et al. conducted five cycles with a 0.5 g/L BSA solution [231] while Xu et al. studied fouling of their blending modified UF photocatalytic membrane reactor using a 1 g/L BSA solution and conducting one cycle also followed by an irradiation step (aiming at degrading the adsorbed proteins from the pores) [369]. The lack of a standard procedure makes it difficult to compare the antifouling performances of a membrane during filtration, in light of existing literature. So, researchers tend to use controls which are the pristine (unmodified) membrane or a commercial membrane possibly of the same composition in terms of main polymer (though difficult to check since actual material compositions are not always revealed by manufacturers) and similar pore size. In order to assess the effect of the modification on fouling mitigation, researchers determine the *FRR*, but also the reversible flux decline ratio (*R_r_*), the irreversible flux decline ratio (*R_ir_*), and the total flux decline ratio (*R_t_*). Their expressions are reminded below:

(1) $$FRR\left(\%\right)=\frac{J_{w,f}}{J_{w,i}}\times 100$$

(2) $$R_{r}\left(\%\right)=\left(\frac{J_{w,f}-J_{foulant,f}}{J_{w,i}}\right)\times 100$$

(3) $$R_{ir}\left(\%\right)=\left(1-\frac{J_{w,f}}{J_{w,i}}\right)\times 100\;$$

(4) $$R_{t}\left(\%\right)=R_{r}+R_{ir}=\left(\frac{J_{w,i}-J_{foulant,f}}{J_{w,i}}\right)\times 100$$

where *J_w,i_* and *J_w,f_* are the initial and final (after membrane cleansing at the end of the cyclic filtration test) pure water flux, respectively, while *J_foulant,f_* represents the flux recorded during the final cycle involving the solution of foulant. If efficient, the modification will have a clear impact on both *FRR* and $R_{ir}$, meaning that some appropriate washing procedure will permit the recovery of the membrane initial permeability as it minimizes irreversible interactions. Then again, however, there is no clear standard procedure for washing the membrane in these tests. Rejections of the foulant material (BSA or others) are also sometimes reported, but the aim of the modification remains to minimize fouling while maintaining other properties. We have discussed in previous sections that the antifouling material affects membrane structure. As a result, it influences both the membrane permeability and the rejection, since parameters such as the pore size, the porosity or the tortuosity associated with the morphology play a major role on the membrane performances. To eliminate these effects and visualize more efficiently the effect of the material on antifouling performances during cyclic filtration, the flux can be normalized using the initial pure water permeability of the membrane [238,365]. This approach also facilitates the comparison with control commercial membranes whose permeability is often different from that of the modified membrane fabricated in the lab. Indeed, the normalized permeability varies in the range 0–1 with 0 indicating total pore blockage, with 1 being the maximum flux recovery ratio (no fouling).

Although BSA dominates, other foulants are used. Humic acid has been employed [43,108,122,142], as well as oil [63,180], extracellular polymeric substances (EPS) [238], diluted commercial milk [106], microalgae (MA) [153,373] or bacteria [123,363]. Microalgae and bacterial solution are particularly appropriate to the performance evaluation of MF membranes, typically prepared by VIPS or TIPS, given their characteristic size. Nevertheless, careful cleaning and disinfection of the equipment has to be performed after bacterial filtration test. Interestingly, despite significantly smaller pore size than the characteristic size of bacteria tested (*Escherichia coli*), VIPS membranes were not permitted to reach 100% rejection, due to the deformability of the cell wall [123]. Thus, one cannot just rely on size-sieving effects in order to achieve safe disinfection of water. Instead, antibacterial agents (positively charged polymers or nanoparticles) would be needed that would kill during filtration such that potential bacteria managing to permeate through the membrane would end up dead.

Different from most studies, some teams have utilized their antifouling membranes to treat more complex feeds, sometimes from natural effluents or wastewater. Notably, Yu et al. treated the supernatant liquor obtained from a sedimentation tank located in a municipal wastewater plant [209]. Xia and Ni used micro-polluted raw water from a river [305], while Yong et al. used oily wastewater from a sewage plant belonging to an oil corporation [142]. Moghadam et al. used EPS, composed of multiple organic substances triggering fouling in membrane bioreactors [238]. The complex nature of the feed made of numerous potential foulants truly challenges the membranes and provides actual evidence of their true potential in real life applications. Nevertheless, this approach remains rare as most tests conducted involve a synthetic feed made of one type of foulant in an aqueous solvent.

Finally, a hybrid static/dynamic test was employed to study biofouling by blood components [41,373]. It consisted of recording the water permeability followed by a long-incubation of the membranes with platelet-poor-plasma (PPP). Then, the permeability was recorded immediately after and before cleaning the membranes. A final water permeability cycle ended the test. The incubation of the membrane with PPP was carried out in a similar way as static adsorption tests, and thus, involved low volume of blood-derivative. Given its composition, PPP has a strong “fouling power”. In addition, because incubation was directly followed by a water permeability test without prior cleaning, this hybrid fouling test designed for biomedical membranes severely challenged the polymer matrix.

### 4.4. Oil Fouling Tests

Oil fouling poses a serious threat to the long-term performance of the membranes. Distinct from biofoulants, oils tend to coalesce and spread across the surface. Since oil repels water, making the surface of the membrane superhydrophilic to prevent oil fouling seems to be the best option. However, this is no easy feat because dissimilar from biofoulants, oils tend to coalesce and spread across the surface. Although it will not form interactions with the hydrated surface of the membrane, the oils would sit on top of it and form an oil layer which hinders the passageways of the water.

Previous studies have placed much emphasis on the wettability tests of the surface which includes, static contact angles of [379]: water in air, water under oil, oil underwater, and sliding contact angle tests in air [380]. For dynamic filtration of oil/water separation, Guo et al. prepared a series of 500 mL feed with 99:1 ratio of water and soybean with 10 mg of sodium dodecyl sulfate (SDS) surfactant [381]. They then performed cyclical filtration of emulsion and water for 18 cycles (9 cycles for oil/water emulsion and 9 cycles of pure water). Amid et al. [263] also evaluated the oil antifouling properties of their modified membranes using three different of olive oil (100 ppm, 150 ppm, and 200 ppm) in the SDS stabilized water-rich emulsions. Other studies have also reported the use of surfactant-stabilized emulsions to test the antifouling properties of membranes [382,383,384]. By using more complex synthetic emulsion feeds, this precedes the development of more effectively robust and highly foulant resistant membranes with wider applicability (e.g., cosmetics and pharmaceutical industries). 

## 5. Conclusions and Future Perspectives

This paper intended to review the recent progress made on the development of antifouling membranes by blending modification, that is, obtained after blending the antifouling additive with a polymer/solvent system before inducing phase separation. We briefly discussed the different phase-inversion processes used to design such membranes, as well as classifying the type of additives (polymer/organic-based, metal-based, ceramic-based, carbon allotrope and porous nanomaterials, and hybrid nanomaterials), examined their effect on fouling and finally introduced common tests carried out by researchers to assess fouling on membranes for wastewater treatment or biomedical-related applications. We saw that the addition of antifouling material to the casting solution in view of improving the antifouling properties is almost always accompanied by an effect on the structure of the membrane due to the change in thermodynamic stability and kinetic of phase-inversion. In the case of LIPS membranes, it often results in the formation of larger macrovoids. In the case of VIPS membrane applied to PVDF (the most used polymer), the additive enables to reduce the size of nodules, while in the case of TIPS, it may affect the dominating phase separation mechanism (i.e., L/L vs. S/L phase separation) especially when nanoparticles are added. Defects and pore blockage are also a commo observation when nanoparticles are added. Thus, changes in permeability and rejection, compared to the pristine (unmodified) membrane is very frequently reported.

Surely, significant progress has been made in the last decade. Yet, after surveying the recent literature, it is clear that efforts still need to be made concerning the following aspects:

Mitigating fouling while maintaining rejection: Thanks to the work of multiple teams worldwide, we have access to a library of materials that efficiently reduce fouling. Nevertheless, the rejection as measured with the pristine membrane is rarely maintained. The blending modified membrane can be seen as a system with distinct retention properties. As such, we may question its domain of application. Can it be used for the same separation or the same treatment as the initial pristine membrane? We believe that researchers should now not just look at the antifouling properties, but instead try to adjust the pore size of the antifouling membrane to the same value as that of the pristine membrane. This means that membrane formation mechanisms should be better controlled (if the antifouling materials accelerates mass transfers during phase-inversion, then one could adjust the non-solvent power of the non-solvent bath by using solvent), and care should be put on fine-tuning the viscosity of the casting solution containing the antifouling material.

The preparation of blending modified membranes in the MF range: LIPS clearly dominates. This has also been the case in the reports published in the past 10 years. This is understandable considering its ease of implementation (in the lab, no expensive equipment is needed except a casting knife to control the initial clearance). However, it leads to UF membranes. Complete wastewater treatment with membrane technology can be achieved providing also the use of smaller (NF) and larger (MF) pore size membranes. Surely, it is very difficult to prepare NF membranes in one step. Moreover, as we have seen, the antifouling material often acted as a pore-former, and it becomes trickier to prepare blending modified membrane for antifouling by phase-inversion. However, how about MF membranes? Both VIPS and TIPS can enable their preparation. The development of blending modified membranes by VIPS has been reported by very few teams including ours. Yet this technique is appropriate to form bi-continuous structures that maintain high flux/mechanical stability and avoid the formation of typical macrovoids (seen in LIPS membranes that weaken the structure). As for the TIPS process, the limitation might be the high temperature of the initial dope that may damage antifouling polymers. However, some nanoparticles have outstanding thermal stability, such that they could be potential candidates for the formation of MF antifouling membranes by blending modification. Too little has been carried out on these aspects and there is room for improvements [23].

The development of blending modified membranes with zwitterionic materials: this class of materials is seen as the latest generation of antifouling materials, providing better stability than PEGylated materials in complex media, and stronger hydration. In recent few years, they have often been used for the surface modification of membranes and dense interfaces to be applied in biomedical devices in contact with biological fluids [385,386,387]. Nevertheless, their use in direct combination with polymers remains limited. The large polarity of the zwitterionic moieties makes it difficult to solubilize them in polymer/solvent systems and prepare homogeneous casting solutions. Yet, addressing solubility issues could further extend the span of applications of blending modified membranes in the biomedical fields (blending membranes for leukodepletion, membranes used as wound-dressings, etc.).

The use of green solvent in the casting solution to form green blending modified membranes: nowadays, the search for green solvents that could be applied to the preparation of phase-inversion membranes is an important direction explored by numerous teams. Although it was beyond the scope of this survey, most papers reviewed here make use of toxic solvents. Thus, not only should we find alternatives to toxic solvents for phase-inversion membranes, but also these greener options should permit the preparation of antifouling membranes in one step. Dimethyl sulfoxide is a greener option and has recently been explored for the making of antifouling membranes by blending modification [304,374], as well as cyrene has been reported in a PES/GO blend [304], but unquestionably a lot more must be achieved to make antifouling membrane production green.

Improving the miscibility of the additives in the polymer matrix and preventing agglomeration: Although physical blending provides a simple approach to modifying both the surface and bulk of the membranes, it is limited by considerable drawbacks such as miscibility issues of some polymeric additives, and agglomeration of inorganic nanoparticles. These problems result in poor dispersion of the additives in the matrix which results in leaching thus, decreasing the antifouling properties of the membranes. In addition, the pores the “leached additives” generate in the polymer matrix albeit, could increase permeability, creates pores on the surface which lowers the rejection, and also induces the formation of large macrovoids along the cross-section which decreases the mechanical strength of the membranes. Moreover, the agglomeration of inorganic additives is found to have induced increased surface roughness which significantly affects the antifouling properties of the membrane as it decreases steric repulsion against foulants. In order to mitigate these problems, recent works have utilized in situ polymerization [372,388,389,390,391,392,393,394,395,396,397,398,399,400,401,402,403], in situ self-assembly of polymeric nanoparticles [404], in situ growth or in situ synthesis [399,405], and in situ generation of nanomaterials [391,406] in the casting solution as strategies to help counter the agglomeration of nanoparticles or nanomaterials in the polymer matrix thus, enhancing the polymer–additive interaction [33]. In order to achieve the latter, the precursors of the nanomaterials must be soluble in the dope casting solution and can react with the non-solvent in the coagulation bath [34,349].

Testing the leachability of inorganic additives: Although tremendous studies have shown the effectiveness of using inorganic nanoparticles in mitigating foulants, it is undeniable that they could still pose massive threats as they could be easily leached out. When this happens, millions of lives could be in danger of metal poisoning. According to previous works [399,405], growth of these nanoparticles in the casting solution could help improve the compatibility of the nanoparticles in the host matrix. However, more studies involving long-term stability are needed in order to test this approach. In addition, leaching tests of these additives should be carried out using sophisticated techniques such as Inductively Coupled Plasma (ICP) Spectroscopy to ensure precise measurements [399].

The need for standard procedures to assess the antifouling properties of membranes: This does not just concern blending modified membranes, but also antifouling membranes prepared by other processes (coating, grafting, etc.). We emphasized some different testing procedures that exist using BSA and reported in the works analyzed for this review. The concentration of the protein, the number of cycles, the duration of these cycles varies from one study to another, or the transmembrane pressure applied such that it is extremely challenging to assess the actual antifouling properties of a membrane. We should be more precise and define some standard testing procedures enabling a fair comparison of the membrane performances. Although this could be a bit tricky given that most of filtration systems in these studies are “in-house”, many parameters would have to depend on the filtration set-up. However, there are still few parameters that could be fixed, and could be established as “protocols” for the antifouling tests such as the concentration of the foulants, and the number of cycles for the “foulant/water” filtration. A study on the domestic wastewater of Shanghai showed that the concentration of total chemical oxygen demand (COD) ranges from 204–440 mg/L (population of 12,000) [407]. Meanwhile, in Indonesia, the COD of greywater wastewater consist of 232–780 mg/L [408]. Considering the COD values of domestic wastewater from these studies, for lab-scale experiments, it would be more logical to set the feed concentration closer to the higher COD recorded to simulate the extreme conditions of wastewater, that is, setting the foulant concentration to 1000 mg/L. In doing so, we could assure that the antifouling properties of the modified membranes could withstand “real-world” conditions. Additionally, in terms of cyclical filtration tests, 3–5 cycles should be carried out (with three repetitions for each condition) so that the stability of the antifouling membranes could be well investigated.

Testing the membranes with more foulants and complex feeds: This again does not just concern blending modified membranes. While surveying the recent literature, we noticed the dominance of one type of feed solution (BSA solution). Testing the antifouling properties with various foulants each time a system is presented would provide supporting evidence of its efficiency to mitigate fouling in different situations. Possibly, natural feeds that combine multiple foulants should be used as well. A few studies made use of complex natural feeds, but more should be carried out in this direction in view of justifying the need for upscaling the production of antifouling membranes at an industrial scale, clearly highlighting their potential range of applications, and showcasing how they can benefit people.

## Figures and Tables

**Table 1:** Polymer/organic-based antifouling additives used in the last 10 years for the blending modification of polymeric membranes.

Polymer/Organic-Based Additives	Materials	Ref.
Hydrophilic or amphiphilic	Poly(ethylene glycol) and its derivatives	[1,41,42,86,87,88,89,90,91,92,93]
	Poly(vinylpyrrolidone)	[43,94,95,96,97]
	Cellulose nanocrystals	[98,99,100,101,102,103]
	Poly(vinyl alcohol)	[104,105,106,107]
	Poly(acrylic acid)	[44,108,109]
	Polydopamine	[110,111,112,113,114]
	Zwitterion/amphiphilic zwitterion	[115,116,117,118,119,120,121,122,123,124]
	Amphiphilic copolymer	[95,125,126,127,128,129,130]
Nature-derived biopolymers	Vanillin	[131,132,133]
	Chitosan	[134,135,136,137,138]
	Chitin	[139,140]
	Lignin	[141,142]
	Lignocellulose	[143]
	Caramel	[144]
	Capsaicin	[145,146]
	Acacia gum	[147]
	Isocyanate	[148,149]
	Organic acids	[45,150,151]
	p-aramid	[152]

**Table 2:** Metal-based antifouling additives used in the last 10 years for the blending modification of polymeric membranes.

Metal-Based Additives	Materials	Ref.
Metallic	Ag and bio-derived Ag	[172,173,174,175,176]
	Au	[177]
	Pd	[178]
Biphasic metals	ZrO_2_	[179,180,181,182]
	ZnO	[183,184,185,186,187,188]
	TiO_2_	[38,58,189,190,191,192,193,194,195,196,197,198,199,200,201,202,203]
	SiO_2_	[204,205,206,207,208,209,210,211,212,213,214,215,216]
	CuO	[186,187,188,217,218]
	Fe_2_O_3_, Fe_3_O_4_	[14,51,205,219,220,221,222,223,224,225,226,227]
	Al_2_O_3_	[55,228,229]
	MXenes	[230]
Bimetallic oxides	Fe-Mn oxide	[231]

**Table 3:** Ceramic-based antifouling additives used in the last 10 years for the blending modification of polymeric membranes.

Ceramic-Based Additives	Materials	Ref.
Nanoclay	Boehmite	[241,242,243]
	Montmorillonite	[244,245,246,247]
	Bentonite	[63,248,249]
	Attapulgite	[250]
	Hydroxyappatite	[251,252,253,254]
	Goethite	[151]
	Cloisite 15A and 30B	[60,255,256]
	Pyrochlores	[255]
	SBA-15	[257,258,259,260,261,262]
	Halloysite	[158,263,264,265,266,267,268,269,270,271,272,273,274,275,276,277,278,279]
	Layered double hydroxide	[64,65,280,281]

**Table 4:** Carbon allotropes and porous nanomaterial as antifouling additives used in the last 10 years for the blending modification of polymeric membranes.

Carbon Allotropes and Porous Nanomaterials	Materials	Ref.
Carbon allotropes	Carbon quantum dot	[286,287]
	Nanodiamond	[72,288,289,290,291,292]
	Carbon nanotubes	[293,294,295,296,297,298,299,300,301,302,303]
	Graphene and its derivatives	[157,304,305,306,307,308,309,310,311,312,313,314,315,316,317,318,319,320,321,322,323,324,325,326,327,328]
Porous nanomaterials	Metal-organic framework	[68,70,329,330,331,332,333,334,335,336,337,338,339,340]
	Zeolites/ZIF	[73,341,342]
	Cyclodextrin	[343]

## Data Availability

Not applicable.

## Abbreviations

BCM	Bamboo cellulose membrane
BSA	Bovine serum albumin
CA	Cellulose acetate
CB	Carboxybetaine
CNC	Cellulose nanocrystal
CND	Carboxylated nanodiamond
DMSO	Dimethyl sulfoxide
DOC	Dissolved organic carbon
DOPA	Dopamine
EPS	Extracellular polymeric substances
FN	Fibrinogen
*FRR*	Flux recovery ratio
GN	Graphene
GO	Graphene oxide
GQDs	Graphene quantum dots
HA	Humic acid
HC	Hydration capacity
HSA	Human serum albumin
LY	Lysozyme
MA	Microalgae
MF	Microfiltration
MMM	Mixed matrix membrane
MPC	2-methacryloyloxyethyl phosphorylcholine
MWCNT	Multiwalled carbon nanotubes
NF	Nanofiltration
OMWCNT	Oxidized multi-wall carbon nanotube
PAA	Poly (acrylic acid)
PACMO	Poly(*N*-acryloylmorpholine)
PAN	Polyacrylonitrile
PANI	Polyaniline
PC	Phosphorylcholine
PE	Polyethylene
PEES	Poly(ether ether sulfone)
PEG	Poly(ethylene glycol)
PEGMA	Poly(ethylene Glycol) Methyl Ether Methacrylate
PEI	Polyetherimide
PES	Poly(ether sulfone)
PLA	Polylactic acid
PP	Polypropylene
PS	Polystyrene
PSBMA	Poly(sulfobetaine methacrylate)
PSf	Polysulfone
PTFE	Polytetrafluoroethylene
PVA	Poly(vinyl alcohol)
PVC	Poly(vinyl chloride)
PVDF	Polyvinylidene fluoride
PVP	Polyvinylpyrrolidone
PWF	Pure water flux
rGO	Reduced graphene oxide
*R_ir_*	Irreversible flux ratio
*R_r_*	Reversible flux ratio
*R_t_*	Total flux ratio
RO	Reverse osmosis
SB	Sulfobetaine
SBAA	Sulfobetaine methacrylamide
SBMA	Sulfobetaine methacrylate
SEM	Scanning electron microscopy
SM	Styrene maleic anhydride
SPANI	Sulfonated polyaniline
UF	Ultrafiltration
WCA	Water contact angle
ZnO-NP	Zinc oxide nanoparticles
ZnO-NR	Zinc oxide nanorods
zP(S-*r*-4VP)	Zwitterionic poly(styrene-*random*-4-vinylpyrridine)
